# Madecassic acid, the contributor to the anti-colitis effect of madecassoside, enhances the shift of Th17 toward Treg cells via the PPAR*γ*/AMPK/ACC1 pathway

**DOI:** 10.1038/cddis.2017.150

**Published:** 2017-03-30

**Authors:** Xiaotian Xu, Yuhui Wang, Zhifeng Wei, Wenhui Wei, Peng Zhao, Bei Tong, Yufeng Xia, Yue Dai

**Affiliations:** 1 Department of Pharmacology of Chinese Materia Medica, China Pharmaceutical University, 24 Tong Jia Xiang, Nanjing 210009, China

## Abstract

The imbalance between Th17 and Treg cells substantially contributes to the intestinal immune disturbance and subsequent tissue injury in ulcerative colitis. The triterpenoid**-**rich fraction of *Centella asiatica* was able to ameliorate dextran sulfate sodium**-**induced colitis in mice. Here we explored its active ingredient and underlying mechanism with a focus on restoring the Th17/Treg balance. The four main triterpenoids occurring in *C. asiatica* were shown to attenuate colitis in mice by oral administration. The most effective ingredient madecassoside lost anti**-**colitis effect when applied topically in the colon, and madecassic acid was recognized to be the active form of madecassoside. Oral administration of madecassic acid decreased the percentage of Th17 cells and downregulated the expression of ROR*γ*t, IL**-**17A, IL**-**17F, IL**-**21 and IL**-**22 and increased the percentage of Treg cells and the expression of Foxp3 and IL**-**10 in the colons of mice with colitis, but it did not affect Th1 and Th2 cells. Under Th17**-**polarizing conditions, madecassic acid downregulated ACC1 expression and enhanced the shift of Th17 cells toward Treg cells, but it did not affect the differentiation of Treg cells under Treg**-**polarizing conditions. Both compound C and AMPK siRNA inhibited the madecassic acid**-**mediated downregulation of ACC1 expression and shift of Th17 cells to Treg cells under Th17**-**polarizing conditions. GW9662, T0070907 and PPAR*γ* siRNA blocked the effect of madecassic acid on AMPK activation, ACC1 expression and shift of Th17 cells to Treg cells. Furthermore, madecassic acid was identified as a PPAR*γ* agonist, as it promoted PPAR*γ* transactivation. The correlation between activation of PPAR*γ* and AMPK, downregulation of ACC1 expression, restoration of Th17/Treg balance and attenuation of colitis by madecassic acid was validated in mice with DSS**-**induced colitis. In conclusion, madecassic acid was the active form of madecassoside in ameliorating colitis by restoring the Th17/Treg balance via regulating the PPAR*γ*/AMPK/ACC1 pathway.

Ulcerative colitis (UC) is a debilitating syndrome characterized by colonic mucosal ulceration, abdominal pain and intestinal barrier dysfunction.^1, 2^ Immune system dysfunction, particularly an imbalance of Th17 cells and Treg cells, contributes substantially to the occurrence and development of UC.^3, 4, 5^ An excess of Th17 cells and insufficient Treg cells result in persistent immune dysfunction and sustained intestinal inflammation.^6, 7, 8^ Therefore, restoring the balance of Th17/Treg cells may be a practical therapeutic strategy for treating UC.

*Centella asiatica* (L.) Urban, a perennial herbaceous plant with pleiotropic bioactivities, mainly consists of pentacyclic triterpenes, including the glycosides madecassoside and asiaticoside as well as their corresponding aglycones madecassic acid and asiatic acid.^9, 10, 11^ Our previous studies demonstrated that the triterpenoid-rich fraction of this herb could ameliorate dextran sulfate sodium (DSS)-induced colitis in mice (unpublished data). Madecassoside, the most abundant triterpene in this herb, was shown to regulate the balance of Th17/Treg cells in a collagen-induced arthritis in rats.^12^ Whether it functions as the primary active ingredient of *C. asiatica* in ameliorating colitis by restoring the Th17/Treg balance remains to be determined.

The balance of Th17/Treg cells can be restored by reducing the generation of Th17 cells, promoting the development of Treg cells and enhancing the phenotypic shift between Th17 and Treg cells.^13, 14^ Accumulative evidence suggests that nuclear receptors, especially peroxisome proliferator-activated receptor *γ* (PPAR*γ*), has a vital role in regulating Th17/Treg balance.^15, 16, 17^ The PPAR*γ* agonists inhibit Th17 cell differentiation in lung myeloid dendritic cells and promote Treg cell differentiation in the white adipose tissue of mice.^18, 19, 20, 21^ Meanwhile, various pentacyclic triterpenes were reported to activate PPAR*γ*.^22, 23^ These findings suggested that the triterpenes in *C. asiatica* might restore the Th17/Treg balance through the PPAR*γ* pathway.

The present study aimed to identify the primary active ingredient of *C. asiatica* and explore its underlying mechanisms for anti**-**UC potential with an emphasis on the Th17/Treg balance.

## Results

### Madecassoside, the main ingredient of *C. asiatica*, attenuated DSS-induced colitis in mice through its aglycone madecassic acid

**Figure 1:**
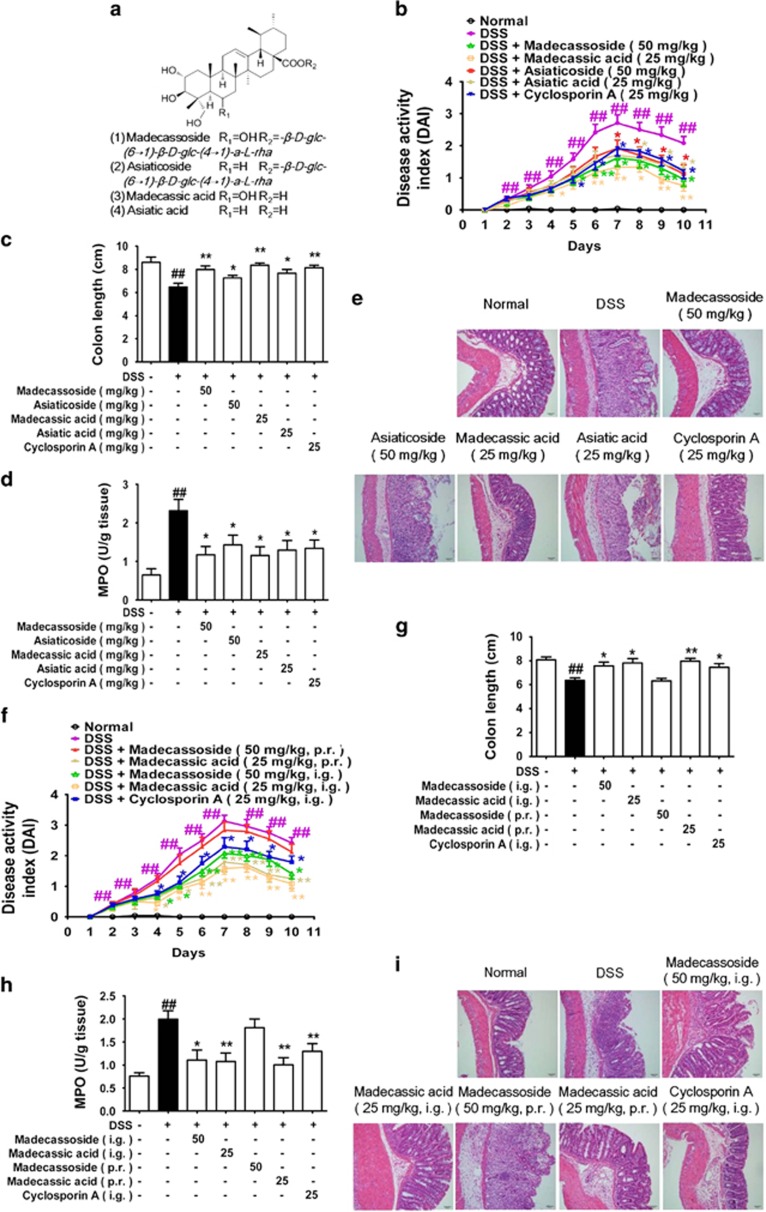
Madecassic acid was the active form of madecassoside in ameliorating DSS-induced colitis in mice. Mice were fed with 2.5% DSS for 7 days and then given normal water for an additional 3 days. Madecassoside (50 mg/kg), asiaticoside (50 mg/kg), madecassic acid (25 mg/kg), asiatic acid (25 mg/kg) and cyclosporin A (25 mg/kg) were orally administered for consecutive 10 days. (**a**) Chemical structures of madecassoside, asiaticoside, madecassic acid and asiatic acid. (**b**) DAI. (**c**) The colon length of each group at day 10. (**d**) The activity of MPO in the colons. (**e**) The epithelial damage, inflammatory cell infiltration and crypt lesions were evaluated by H&E staining. (**f**) Mice were rectally administered test compounds for 10 days. DAI. (**g**) Colon length. (**h**) MPO activity. (**i**) H&E staining. The data were expressed as means±S.E.M., *n*=5–8. ^##^*P*<0.01 *versus* normal group; *P<0.05, **P<0.01 *versus* DSS group

Drinking DSS induces severe colitis in mice.^24^ To clarify the active ingredients for the anti**-**colitis effect of *C. asiatica*, four major pentacyclic triterpenes isolated from the plant (madecassoside, asiaticoside, madecassic acid and asiatic acid, Figure 1a) and cyclosporin A were orally administered to DSS**-**treated mice for 10 days. Madecassoside (50 mg/kg) and madecassic acid (25 mg/kg) were shown to be more effective than asiaticoside (50 mg/kg) and asiatic acid (25 mg/kg), respectively, as evidenced by decreasing disease activity index (DAI) scores (Figure 1b), reducing colon shortening (Figure 1c), lowering myeloperoxidase (MPO) activity (Figure 1d) and attenuating pathological injuries of colonic tissues (Figure 1e and Supplementary Figure S1). It appeared that pentacyclic triterpenes with a hydroxyl group were superior to those without a hydroxyl group in ameliorating colitis in mice.

Madecassoside will rapidly metabolize into its aglycone madecassic acid in the small intestine after oral administration. It was necessary to identify the efficient form of madecassoside for attenuating colitis. Our data showed that intra**-**rectal administration of madecassic acid (25 mg/kg) effectively ameliorated colitis in mice, as confirmed by reducing DAI scores (Figure 1f), protecting against colon shortening (Figure 1g), decreasing MPO activity (Figure 1h) and attenuating pathological lesions (Figure 1i and Supplementary Figure S1). In contrast, madecassoside (50 mg/kg) per rectum failed to protect against pathological injury in the colons of mice. These findings revealed that the primary active ingredient madecassoside acted through the intestinal metabolite madecassic acid in ameliorating colitis in mice.

### Madecassic acid restored the Th17/Treg balance in mice with DSS-induced colitis

**Figure 2:**
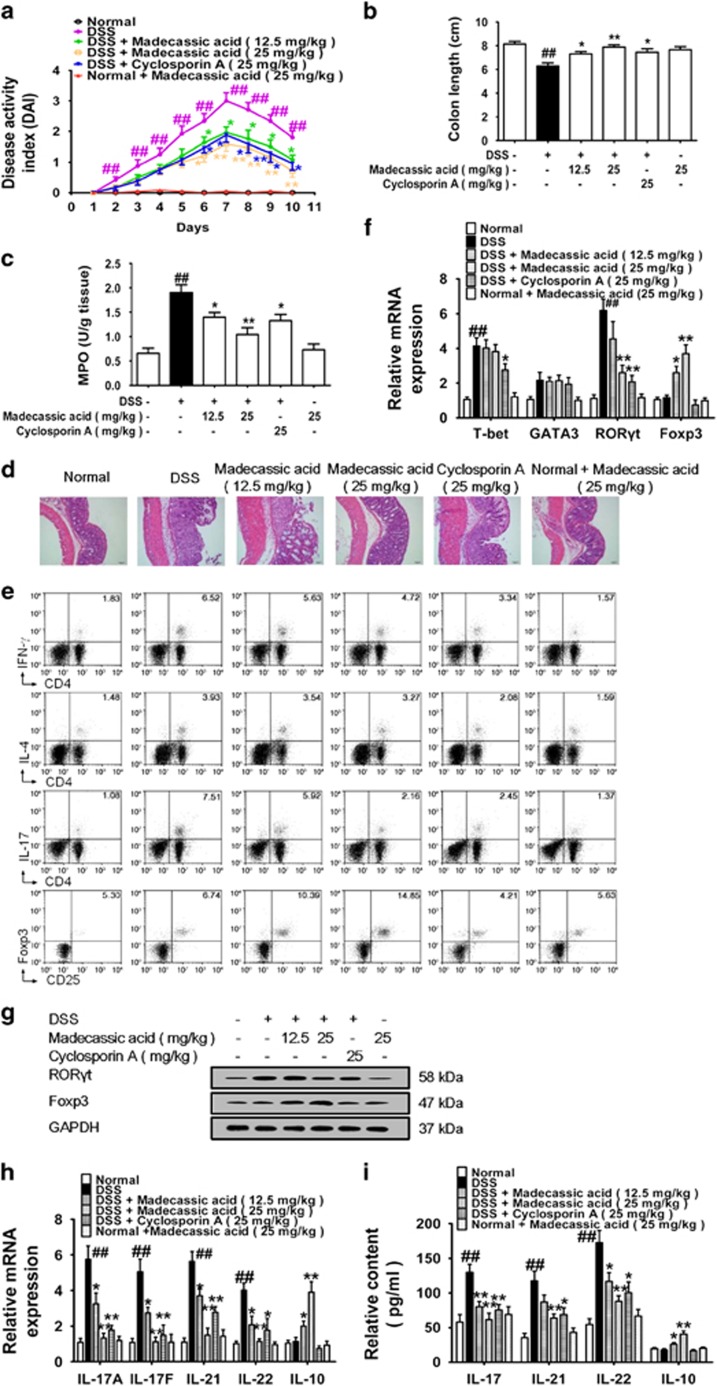
Madecassic acid restored the T helper type 17/regulatory T cell balance in mice with DSS-induced colitis. Mice were fed with 2.5% DSS for 7 days and then given normal water for 3 days. Madecassic acid (12.5, 25 mg/kg) and cyclosporin A (25 mg/kg) were orally administered for 10 days. (**a**) DAI. (**b**) Colon length. (**c**) MPO activity. (**d**) H&E staining. (**e**) The proportions of CD4^+^ IFN**-***γ*^+^ T cells, CD4^+^IL**-**4^+^ T cells, CD4^+^IL**-**17^+^ T cells and CD4^+^CD25^+^Foxp3^+^ T cells were detected by flow cytometry. (**f** and **g**) The mRNA and protein levels of transcription factors T**-**bet, GATA**-**3, ROR*γ*t and Foxp3 in the colons were evaluated by real**-**time PCR and western blot. (**h** and **i**) The levels of IL**-**17A, IL**-**17F, IL**-**21, IL**-**22 and IL**-**10 were assessed by enzyme-linked immunosorbent assay and real**-**time PCR. GAPDH was used as a cytoplasm marker. The data were expressed as means±S.E.M., *n*=5–8. ^##^*P*<0.01 *versus* normal group; *P<0.05, **P<0.01 *versus* DSS group

Madecassic acid (12.5, 25 mg/kg) conferred protection against DSS**-**treated colitis in mice (Figures 2a–d and Supplementary Figure S1). DSS**-**treated mice showed higher percentages of CD4^+^IFN**-***γ*^+^ T cells and CD4^+^IL**-**17^+^ T cells but not CD4^+^IL**-**4^+^ T cells or CD4^+^CD25^+^Foxp3^+^ T cells in the colonic lamina propria. Madecassic acid considerably decreased the percentage of CD4^+^IL**-**17^+^ T cells and increased the percentage of CD4^+^CD25^+^Foxp3^+^ T cells without marked effects on the percentages of CD4^+^IFN**-***γ*^+^ and CD4^+^IL**-**4^+^ T cells (Figure 2e and Supplementary Figure S1). Noteworthy, the ratio of Th17/Treg cells was 0.33 in the normal group, and it climbed to 1.15 in the model group (Figure 2e and Supplementary Figure S1). Madecassic acid (12.5, 25 mg/kg) decreased the ratio of Th17/Treg cells to 0.46 and 0.34, respectively. These data suggested that madecassic acid could restore the balance of Th17/Treg cells in the colons. Consistently, madecassic acid (25 mg/kg) reduced the expression of Th17 cell transcription factor ROR*γ*t, it (12.5, 25 mg/kg) enhanced the expression of Treg cell transcription factor Foxp3, but it did not affect the expression of T**-**bet and GATA**-**3 (Figures 2f, g and Supplementary Figure S1). Additionally, madecassic acid (12.5, 25 mg/kg) downregulated the expression of Th17 cell**-**specific cytokines, while upregulated the expression of Treg cell**-**specific cytokine IL**-**10 in the colons (Figures 2h and i). Taken together, madecassic acid alleviated DSS**-**induced colitis in mice by restoring the balance of Th17/Treg cells in the colon.

### Madecassic acid restored the Th17/Treg balance by enhancing the shift of Th17 toward Treg cells

**Figure 3:**
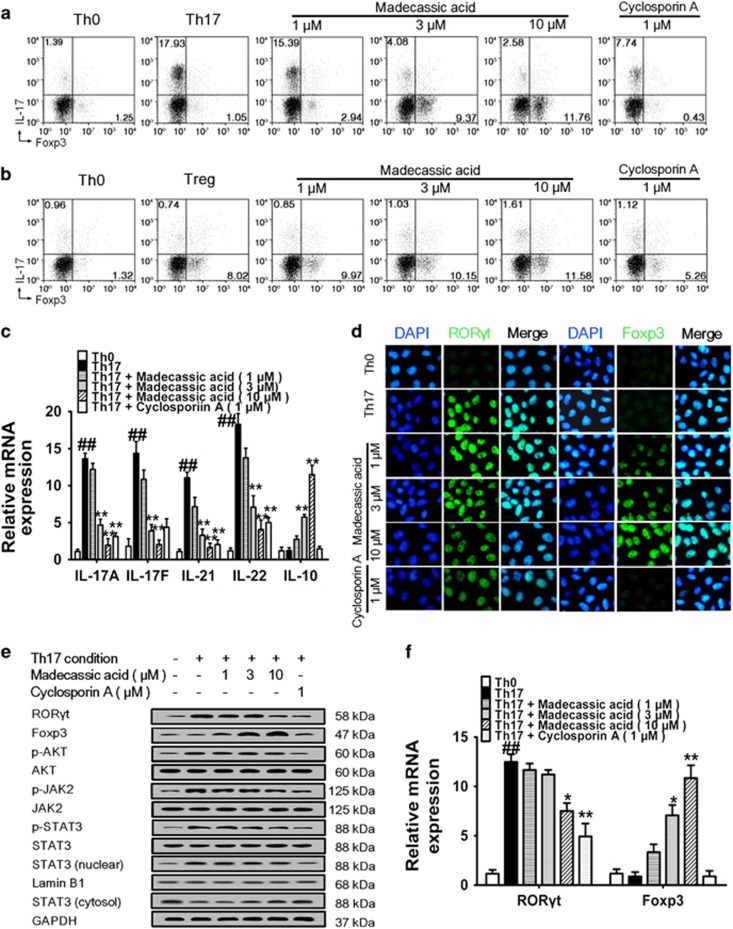
Madecassic acid restored the T helper type 17/regulatory T cell (Th17/Treg) balance by enhancing the shift of of Th17 toward Treg cells. (**a**) Naive T cells were differentiated under Th17**-**inducing conditions for 4 days in the presence of madecassic acid (1, 3, 10 *μ*M) or a control. The proportions of CD4^+^IL**-**17^+^ T cells and CD4^+^Foxp3^+^ T cells were gated by flow cytometry. (**b**) Naive T cells were differentiated under Treg**-**inducing conditions for 4 days in the presence or absence of madecassic acid (1, 3, 10 *μ*M). The proportions of CD4^+^IL**-**17^+^ T cells and CD4^+^Foxp3^+^ T cells were gated by flow cytometry. (**c**) Naive T cells were cultured with madecassic acid (1, 3, 10 *μ*M) under Th17**-**inducing conditions for 4 days. The mRNA levels of IL**-**17A, IL**-**17F, IL**-**21, IL**-**22 and IL**-**10 were measured by real**-**time PCR. (**d**) Naive T cells were cultured with madecassic acid (1, 3, 10 *μ*M) under Th17**-**inducing conditions for 4 days. The expression of ROR*γ*t and Foxp3 under Th17**-**polarizing conditions was measured by immunofluorescence. (**e**) Naive T cells were cultured with madecassic acid (1, 3, 10 *μ*M) under Th17**-**inducing conditions for 4 days. The protein expression of ROR*γ*t and Foxp3, as well as Th17**-**associated transducers p**-**AKT, AKT, p**-**STAT3, STAT3, p**-**JAK2 and JAK was examined by western blot. (**f)** Naive T cells were cultured with madecassic acid (1, 3, 10 *μ*M) under Th17**-**inducing conditions for 4 days. The mRNA expression of ROR*γ*t and Foxp3 was analyzed by real**-**time PCR. GAPDH was used as a cytoplasm marker; Lamin B1 was used as a nuclear marker. The data were expressed as means±S.E.M., *n*=3. ^#^*P*<0.05, ^##^*P*<0.01 *versus* Th0 group; *P<0.05, **P<0.01 *versus* Th17 group

To recognize how madecassic acid restores the Th17/Treg balance, naive mouse CD4^+^ T cells were cultured under Th17**-** or Treg**-**polarizing conditions.^25, 26^ Madecassic acid (3, 10 *μ*M) decreased the percentage of CD4^+^IL**-**17^+^ T cells but increased the percentage of CD4^+^Foxp3^+^ T cells under Th17**-**polarizing conditions (Figure 3a and Supplementary Figure S1). But it showed very weak effect on the percentages of CD4^+^IL**-**17^+^ T cells and CD4^+^Foxp3^+^ T cells under Treg**-**polarizing conditions (Figure 3b and Supplementary Figure S1). Consistently, madecassic acid (3, 10 *μ*M) downregulated the expression of Th17 cell**-**specific cytokines and upregulated the expression of IL**-**10 under Th17**-**polarizing conditions (Figure 3c), but it did not affect the expression of IL**-**10 under Treg**-**polarizing conditions (Supplementary Figure S2a). Consequently, we postulated that madecassic acid might induce the shift of Th17 toward Treg cells under Th17**-**polarizing conditions instead of directly enhancing Treg differentiation.

To recognize whether the madecassic acid**-**induced shift of Th17 toward Treg cells was achieved by inhibiting conventional Th17 cell differentiation pathways, we investigated the effect of madecassic acid on Th17 and Treg transcription factors and differentiation**-**associated signal transducers under Th17**-**polarizing conditions and in DSS**-**induced mice. Madecassic acid (3 *μ*M) showed weak inhibition on the expression of ROR*γ*t but obviously enriched the expression of Foxp3 under Th17**-**polarizing conditions (Figures 3d–f and Supplementary Figure S1), implying that madecassic acid promoted the shift of Th17 toward Treg cells by inducing Foxp3 expression instead of reducing ROR*γ*t expression. In contrast, the expression of Foxp3 was not affected by madecassic acid under Treg**-**polarizing conditions (Supplementary Figure S2b). Furthermore, madecassic acid only weakly inhibited the phosphorylation of STAT3, JAK2, AKT and translocation of STAT3 under Th17**-**polarizing conditions (Figure 3e and Supplementary Figure S1), implying that madecassic acid-induced shift of Th17 toward Treg cells might not be through the inhibition of conventional Th17 cell differentiation pathways. Similarly, madecassic acid (12.5, 25 mg/kg) showed a little inhibition on the phosphorylation of STAT3, JAK2 and AKT in the colons of DSS**-**induced mice (Supplementary Figure S2d). The phosphorylation of Treg cell activation**-**associated signal transducers STAT5 and Smad3 were not affected by madecassic acid under Treg**-**polarizing conditions and in DSS**-**induced mice (Supplementary Figures S2c and d). These findings indicated that madecassic acid might not affect the activation of STAT3, JAK2 and AKT in the shift of Th17 toward Treg cells.

### Madecassic acid facilitated the shift of Th17 toward Treg cells by suppressing the acetyl CoA carboxylase (ACC1) expression

**Figure 4:**
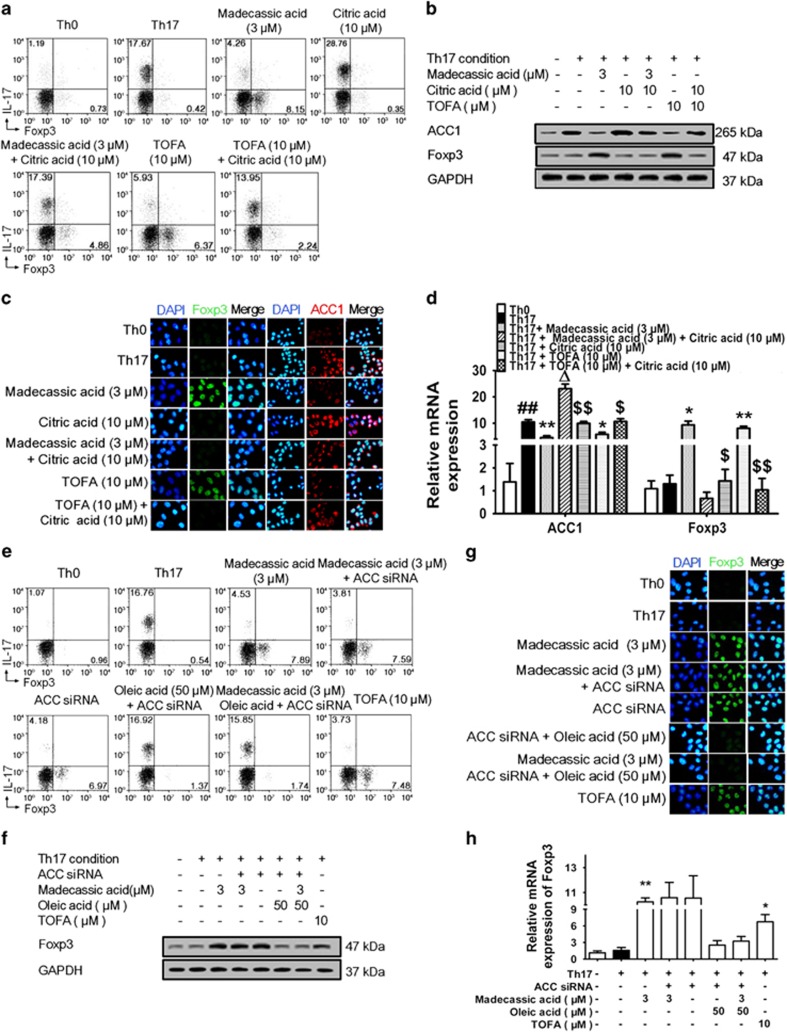
Madecassic acid facilitated the shift of T helper type 17 (Th17) toward regulatory T cells by inhibiting ACC1 expression. (**a**) Naive T cells were cultured with madecassic acid (3 *μ*M) and ACC1 activator citric acid (10 *μ*M) under Th17**-**inducing conditions for 4 days. The proportions of CD4^+^IL**-**17^+^ T cells and CD4^+^Foxp3^+^ T cells were detected by flow cytometry. (**b**–**d**) Naive T cells were cultured with madecassic acid (3 *μ*M) and ACC1 activator citric acid (10 *μ*M) under Th17**-**inducing conditions for 4 days. The mRNA and protein expression of Foxp3 and ACC1 was analyzed by western blot, immunofluorescence and real**-**time PCR. (**e**) Naive T cells were cultured with madecassic acid (3 *μ*M), oleic acid (50 *μ*M) and ACC1 siRNA under Th17**-**polarizing conditions for 4 days. The proportions of CD4^+^IL**-**17^+^ T cells and CD4^+^Foxp3^+^ T cells were detected by flow cytometry. (**f**–**h**) Naive T cells were cultured with madecassic acid (3 *μ*M), oleic acid (50 *μ*M) and ACC1 siRNA under Th17**-**polarizing conditions for 4 days. The relative expression of Foxp3 was analyzed by western blot, immunofluorescence and real**-**time PCR. GAPDH was used as a cytoplasm marker. The data were expressed as means±S.E.M., *n*=3. ^#^*P*<0.05, ^##^*P*<0.01 *versus* Th0 group; *P<0.05, **P<0.01 *versus* Th17 group; Δ*P*<0.05 *versus* Th17 group; ^$^*P*<0.05, ^$$^*P*<0.01 *versus* citric acid group

ACC1, the rate**-**limiting enzyme in fatty acid synthesis that catalyzes the carboxylation of acetyl**-**CoA to malonyl**-**CoA, enhances and inhibits the polarization of Th17 and Treg cells, respectively.^27, 28^ To identify whether the madecassic acid**-**induced shift of Th17 toward Treg cells was related to ACC1 inhibition, we evaluated its effect on ACC1 expression. Madecassic acid (3, 10 *μ*M) downregulated ACC1 expression under Th17**-**polarizing conditions (Supplementary Figure S3a), and oral madecassic acid (12.5, 25 mg/kg) suppressed the ACC1 expression in the colons of DSS**-**treated mice (Supplementary Figure S3b). In addition, the shift of Th17 toward Treg cells induced by madecassic acid was diminished by ACC1 activator citric acid under Th17**-**polarizing conditions (Figure 4a and Supplementary Figure S1). Consistently, citric acid reversed the madecassic acid**-**induced downregulation of Th17 cell**-**specific cytokines expression, the upregulation of IL**-**10 expression (Supplementary Figure S3c) and the enhancement of Foxp3 expression under Th17**-**polarizing conditions (Figures 4b–d and Supplementary Figure S1). The suppression of ACC1 expression by madecassic acid was also diminished by citric acid (Figures 4b–d and Supplementary Figure S1). These findings suggested that madecassic acid enhanced the shift of Th17 toward Treg cells by suppressing the ACC1 expression.

To identify whether the shift of Th17 toward Treg cells induced by madecassic acid take place through the downregulation of ACC1 catalytic products, naive CD4^+^ T cells were subjected to ACC1 depletion or treated with oleic acid (an ACC1 catalytic product) under Th17**-**polarizing conditions. Interestingly, the shift of Th17 toward Treg cells by madecassic acid was not affected by ACC1 depletion but inhibited by oleic acid (Figure 4e and Supplementary Figure S1). In addition, madecassic acid**-**induced decrease in Th17 cell**-**specific cytokines expression and increase in IL**-**10 expression were inhibited by oleic acid under ACC1 depletion (Supplementary Figure S3d). The high level of Foxp3 expression induced by madecassic acid was also blunted by oleic acid (Figures 4f–h and Supplementary Figure S1). These findings suggested that madecassic acid shifted Th17 toward Treg cells through downregulation of ACC1 expression.

### Madecassic acid inhibited ACC1 expression and shifted Th17 toward Treg cells by activating adenosine monophosphate-activated protein kinase (AMPK)

**Figure 5:**
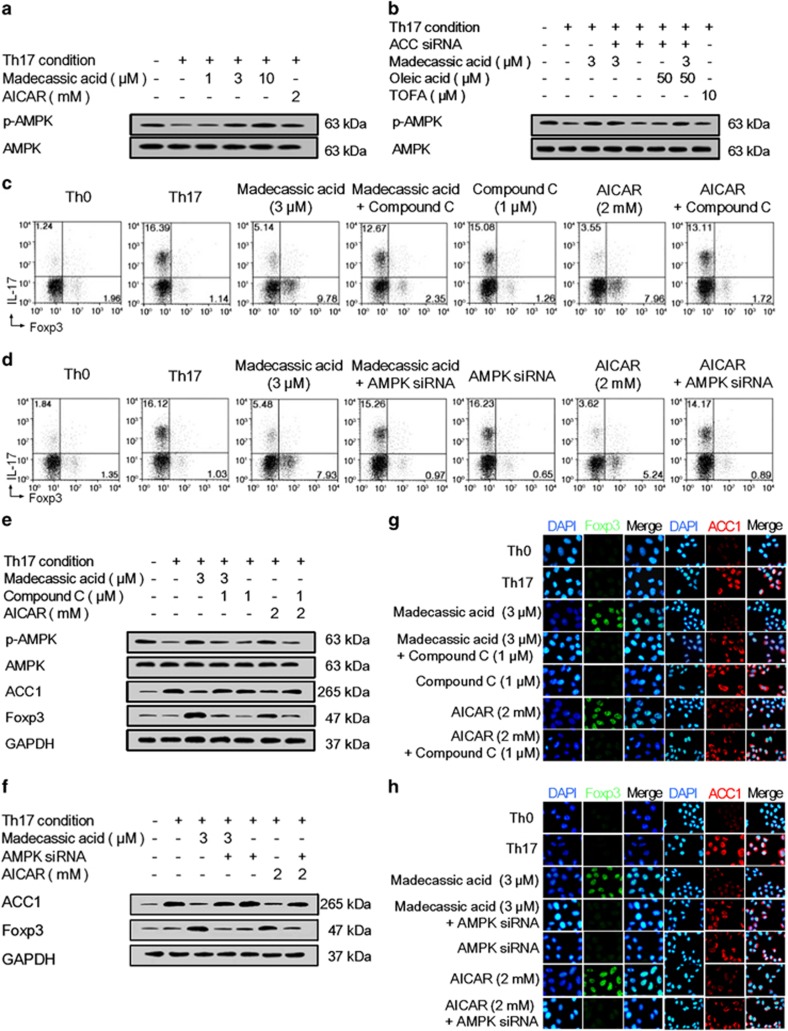
Madecassic acid regulated ACC1-mediated shift of T helper type 17 (Th17) toward regulatory T cells via the activation of AMPK. Naive T cells were cultured with or without compound C and AMPK siRNA under Th17**-**inducing conditions in the presence of madecassic acid for 4 days. (**a**) Naive T cells were treated with madecassic acid (1, 3, 10 *μ*M) under Th17**-**inducing conditions for 4 days. The protein expression of p**-**AMPK was analyzed by western blot. (**b**) Naive T cells were cultured with madecassic acid (3 *μ*M), oleic acid (50 *μ*M) and ACC1 siRNA under Th17**-**polarizing conditions for 4 days. The protein expression of p**-**AMPK was measured by western blot. (**c** and **d**) Naive T cells were cultured with madecassic acid (3 *μ*M), compound C (1 *μ*M) and AMPK siRNA under Th17**-**polarizing conditions for 4 days. The proportions of CD4^+^IL**-**17^+^ T cells and CD4^+^Foxp3^+^ T cells were evaluated by flow cytometry. (**e**) Naive T cells were cultured with madecassic acid (3 *μ*M) and compound C (1 *μ*M) under Th17**-**polarizing conditions for 4 days. The relative expression of p**-**AMPK, AMPK, ACC1 and Foxp3 was analyzed by western blot. (**f**) Naive T cells were cultured with madecassic acid (3 *μ*M) and AMPK siRNA under Th17**-**polarizing conditions for 4 days. The relative expression of ACC1 and Foxp3 was analyzed by western blot. (**g**) Naive T cells were cultured with madecassic acid (3 *μ*M) and compound C (1 *μ*M) under Th17**-**polarizing conditions for 4 days. The relative expression of ACC1 and Foxp3 was analyzed by immunofluorescence. (**h**) Naive T cells were cultured with madecassic acid (3 *μ*M) and AMPK siRNA under Th17**-**polarizing conditions for 4 days. The relative expression of ACC1 and Foxp3 was analyzed by immunofluorescence. GAPDH was used as a cytoplasm marker. The data were expressed as the means±S.E.M., *n*=3. ^#^*P*<0.05, ^##^*P*<0.01 *versus* Th0 group; *P<0.05, **P<0.01 *versus* Th17 group; ^$^*P*<0.05, ^$$^*P*<0.01 *versus* madecassic acid group

ACC1**-**mediated fatty acid synthesis can be inhibited by the activation of AMPK, a cellular energy sensor.^29, 30^ Madecassic acid (3, 10 *μ*M) enhanced the phosphorylation of AMPK under Th17**-**polarizing conditions (Figure 5a and Supplementary Figure S1) and in the colons of DSS**-**induced mice (Supplementary Figure S3b). The inhibitory effect of madecassic acid on ACC1 expression was weakened by AMPK antagonist compound C and siRNA under Th17**-**polarizing conditions (Figures 5e and f). In contrast, madecassic acid**-**induced activation of AMPK was not affected by ACC1 siRNA or oleic acid (Figure 5b and Supplementary Figure S1), suggesting that madecassic acid suppressed the ACC1 expression through the activation of AMPK. Furthermore, both compound C and AMPK siRNA inhibited madecassic acid**-**induced shift of Th17 toward Treg cells (Figures 5c, d and Supplementary Figure S1), regulation of Th17 cell**-**specific cytokines and IL**-**10 expression (Supplementary Figures S3e and f), the increase in Foxp3 expression and the decrease in ACC1 expression (Figures 5e–h and Supplementary Figure S1). These findings suggested that madecassic acid enhanced the shift of Th17 toward Treg cells by downregulating the ACC1 expression via activation of AMPK.

### Madecassic acid promoted AMPK activation in the shift of Th17 toward Treg cells by activating PPAR*γ*

**Figure 6:**
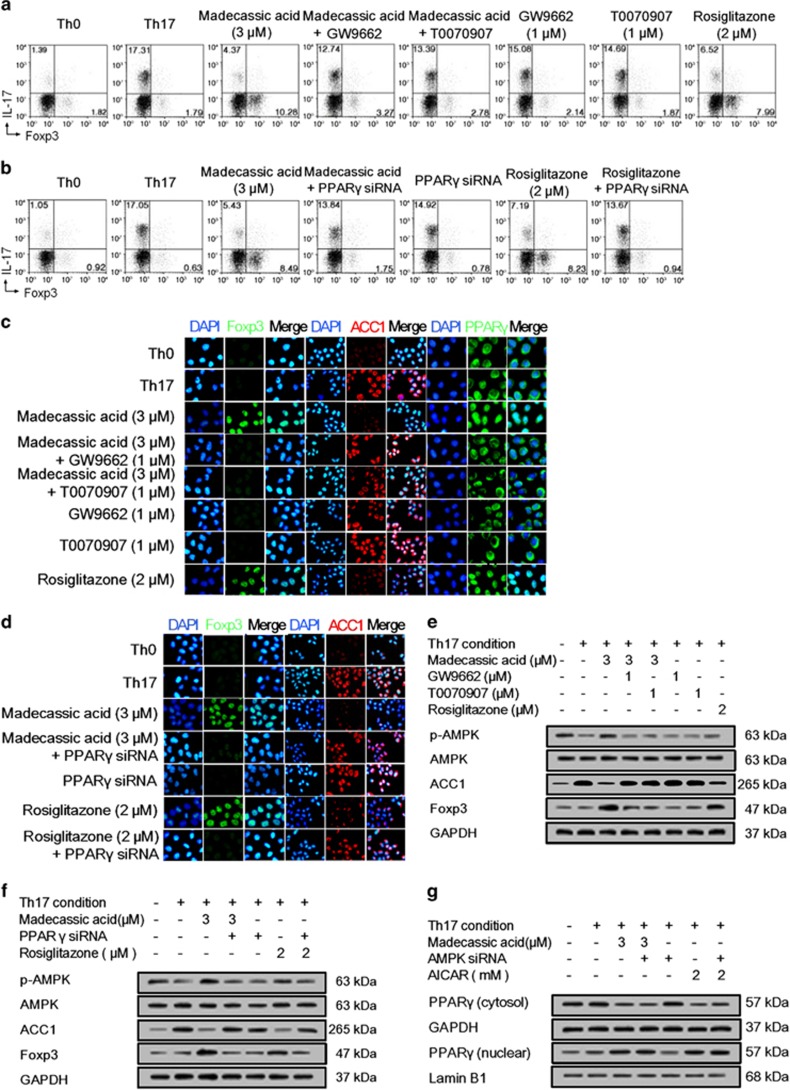
Madecassic acid regulated AMPK/ACC1 via activating PPAR*γ* in the shift of T helper type 17 (Th17) toward regulatory T cells. Naive T cells were treated with GW9662 (1 *μ*M), T0070907 (1 *μ*M) and PPAR*γ* siRNA under Th17**-**polarizing conditions for 4 days in the presence of madecassic acid (3 *μ*M). (**a**) Naive T cells were treated d with GW9662 (1 *μ*M) and T0070907 (1 *μ*M) under Th17**-**polarizing conditions for 4 days in the presence of madecassic acid (3 *μ*M). The proportions of CD4^+^IL**-**17^+^ T cells and CD4^+^Foxp3^+^ T cells were evaluated by flow cytometry. (**b**) Naive T cells were treated with madecassic acid (3 *μ*M) and PPAR*γ* siRNA under Th17**-**polarizing conditions for 4 days. The proportions of CD4^+^IL**-**17^+^ T cells and CD4^+^Foxp3^+^ T cells were evaluated by flow cytometry. (**c**) The relative expression of PPAR*γ*, ACC1 and Foxp3 was evaluated by immunofluorescence in the presence of madecassic acid (3 *μ*M), GW9662 (1 *μ*M) and T0070907 (1 *μ*M). (**d**) The relative expression of ACC1 and Foxp3 was evaluated by immunofluorescence in the presence of madecassic acid (3 *μ*M) and PPAR*γ* siRNA. (**e**) Naive T cells were treated with GW9662 (1 *μ*M) and T0070907 (1 *μ*M) under Th17**-**polarizing conditions for 4 days in the presence of madecassic acid (3 *μ*M). The relative expression of p**-**AMPK, ACC1 and Foxp3 was evaluated by western blot. (**f**) Naive T cells were treated with madecassic acid (3 *μ*M) and PPAR*γ* siRNA under Th17**-**polarizing conditions for 4 days. The relative expression of p**-**AMPK, ACC1 and Foxp3 was evaluated by western blot. (**g**) Naive T cells were treated with madecassic acid (3 *μ*M) and AMPK siRNA under Th17**-**polarizing conditions for 4 days. The relative expression of cytosolic and nuclear PPAR*γ* was measured by western blot. GAPDH was used as a cytoplasm marker; Lamin B1 was used as a nuclear marker. All data were expressed as means±S.E.M., *n*=3. ^#^*P*<0.05, ^##^*P*<0.01 *versus* Th0 group; *P<0.05, **P<0.01 *versus* Th17 group; ^$^*P*<0.05, ^$$^*P*<0.01 *versus* madecassic acid group

Madecassic acid (3, 10 *μ*M) promoted the translocation of PPAR*γ* from the cytoplasm to the nucleus under Th17**-**polarizing conditions (Supplementary Figure S4a). It also promoted PPAR*γ* nuclear translocation in the colons of DSS**-**induced mice (Supplementary Figure S4b). To verify the key role that PPAR*γ* has in the madecassic acid**-**induced shift of Th17 toward Treg cells under Th17**-**polarizing conditions, PPAR*γ* antagonists GW9662 and T0070907 as well as PPAR*γ* siRNA were used. The results showed that either pharmacological antagonism or knockdown of PPAR*γ* inhibited madecassic acid**-**induced shift of Th17 toward Treg cells (Figures 6a and b) and regulation of Th17 cell**-**specific cytokines and IL**-**10 expression (Supplementary Figures S4c and d) and Foxp3 expression (Figures 6c–f and Supplementary Figure S1). These data revealed that madecassic acid promoted the shift of Th17 toward Treg cells by activating PPAR*γ*.

Further studies showed that the madecassic acid**-**induced AMPK activation and ACC1 inhibition were inhibited by GW9662, T0070907 and PPAR*γ* siRNA (Figures 6c–f and Supplementary Figure S1) Conversely, neither pharmacological antagonism nor knockdown of AMPK or ACC1 affected the translocation of PPAR*γ* induced by madecassic acid (Figure 6g and Supplementary Figures S1 and S4e–g). These findings revealed that PPAR*γ* mediated the activation of AMPK and subsequent inhibition of ACC1 expression induced by madecassic acid in the shift of Th17 toward Treg cells.

### Madecassic acid acted as a PPAR*γ* agonist

**Figure 7:**
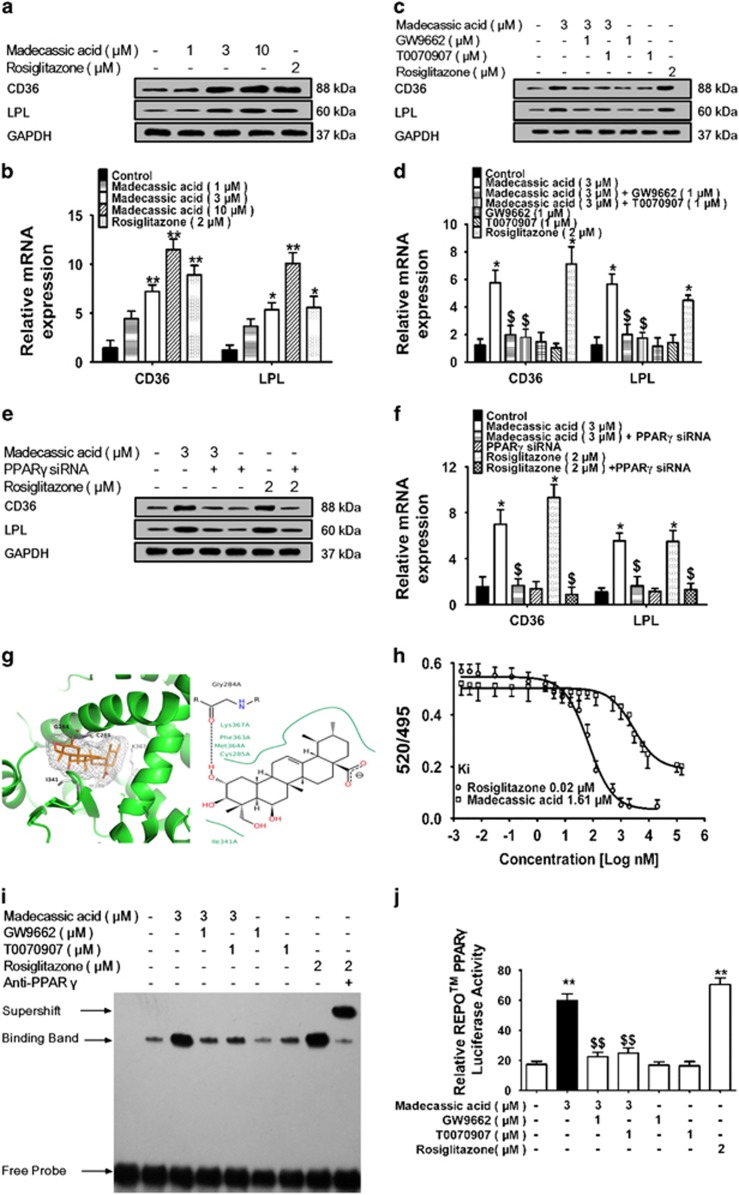
Madecassic acid as a PPAR*γ* agonist. (**a** and **b**) EL**-**4 cells were treated with madecassic acid (1, 3, 10 *μ*M) and rosiglitazone (2 *μ*M) for 24 h. The expression of the PPAR*γ***-**responsive genes CD36 and LPL was quantified by western blot and real**-**time PCR. (**c** and **d**) EL**-**4 cells were treated with madecassic acid (3 *μ*M), GW9662 (1 *μ*M) and T0070907 (1 *μ*M) for 24 h. The expression of CD36 and LPL was measured by western blot and real**-**time PCR. (**e** and **f**) EL**-**4 cells were treated with madecassic acid (3 *μ*M) and PPAR*γ* siRNA for 24 h. The expression levels of CD36 and LPL were measured by western blot and real**-**time PCR. (**g**) The docking between madecassic acid and PPAR*γ***-**LBD in EL**-**4 cells. (**h**) Binding of madecassic acid to PPAR*γ***-**LBD in a competitive TR**-**FRET assay. (**i**) The effect of madecassic acid on the nuclear accumulation of PPAR*γ* in EL**-**4 cells was determined by an EMSA in EL**-**4 cells. (**j**) The effect of madecassic acid on PPAR*γ***-**dependent transactivation in EL**-**4 cells was assayed with a luciferase reporter gene assay. GAPDH was used as a cytoplasmic marker. The data were expressed as means±S.E.M., *n*=3. *P<0.05, **P<0.01 *versus* control group, ^$^*P*<0.05, ^$$^*P*<0.01 *versus* madecassic acid group

Madecassic acid (3, 10 *μ*M) elevated the expression of PPAR*γ***-**responsive genes fatty acid translocase (CD36) and lipoprotein lipase (LPL) in T lymphocytes (EL**-**4 cells) (Figures 7a, b and Supplementary Figure S1) without marked effect on PPAR*α***-** and PPAR*β***-**responsive genes (Supplementary Figure S5a). Madecassic acid (12.5, 25 mg/kg) also induced the expression of CD36 and LPL in the colons of DSS**-**induced mice (Supplementary Figures S5b and c). These data implied that madecassic acid selectively promoted the expression of PPAR*γ***-**responsive genes. GW9662, T0070907 and PPAR*γ* siRNA inhibited the effect of madecassic acid on CD36 and LPL expression (Figures 7c–f and Supplementary Figure S1), suggesting that madecassic acid enhanced CD36 and LPL expression in a PPAR*γ***-**dependent manner. To determine whether the increased CD36 and LPL expression induced by madecassic acid was a result of *de novo* RNA synthesis or protein synthesis, EL**-**4 cells were pretreated with RNA polymerase inhibitor actinomycin D (Act**-**D) or protein synthesis inhibitor cycloheximide (CHX). Both Act**-**D and CHX almost completely abolished the madecassic acid**-**induced increase in the CD36 and LPL expression (Supplementary Figures S5d–f), indicating that the effect of madecassic acid on PPAR*γ***-**responsive genes depended on both RNA and protein synthesis. An in**-**depth analysis of molecular operating environment (MOE) docking module showed that madecassic acid extended deep into the PPAR*γ***-**ligand binding domain (LBD) and interacted with hydrophobic residues at Gly 284, where the H**-**bonds improved the binding affinity of madecassic acid to PPAR*γ* (Figure 7g). A time**-**resolved fluorescence resonance energy transfer (TR**-**FRET) assay further demonstrated that madecassic acid bounded to PPAR*γ* with a kinetic inhibition constant (*K*_i_) of 1.61 *μ*M (Figure 7h). An electrophoretic mobility shift assay (EMSA) was performed to verify that madecassic acid indeed induced the binding of PPAR*γ* to the PPAR response element (PPRE) sequence, which could be diminished by GW9662 and T0070907 (Figure 7i). A luciferase reporter assay showed that madecassic acid markedly enhanced PPRE reporter activity in EL**-**4 cells, which was reduced by GW9662 and T0070907 (Figure 7j). These data suggested that madecassic acid was a potential agonist of PPAR*γ*.

### Madecassic acid recovered Th17/Treg balance in mice with DSS-induced colitis through PPAR*γ*/AMPK/ACC1 pathway

**Figure 8:**
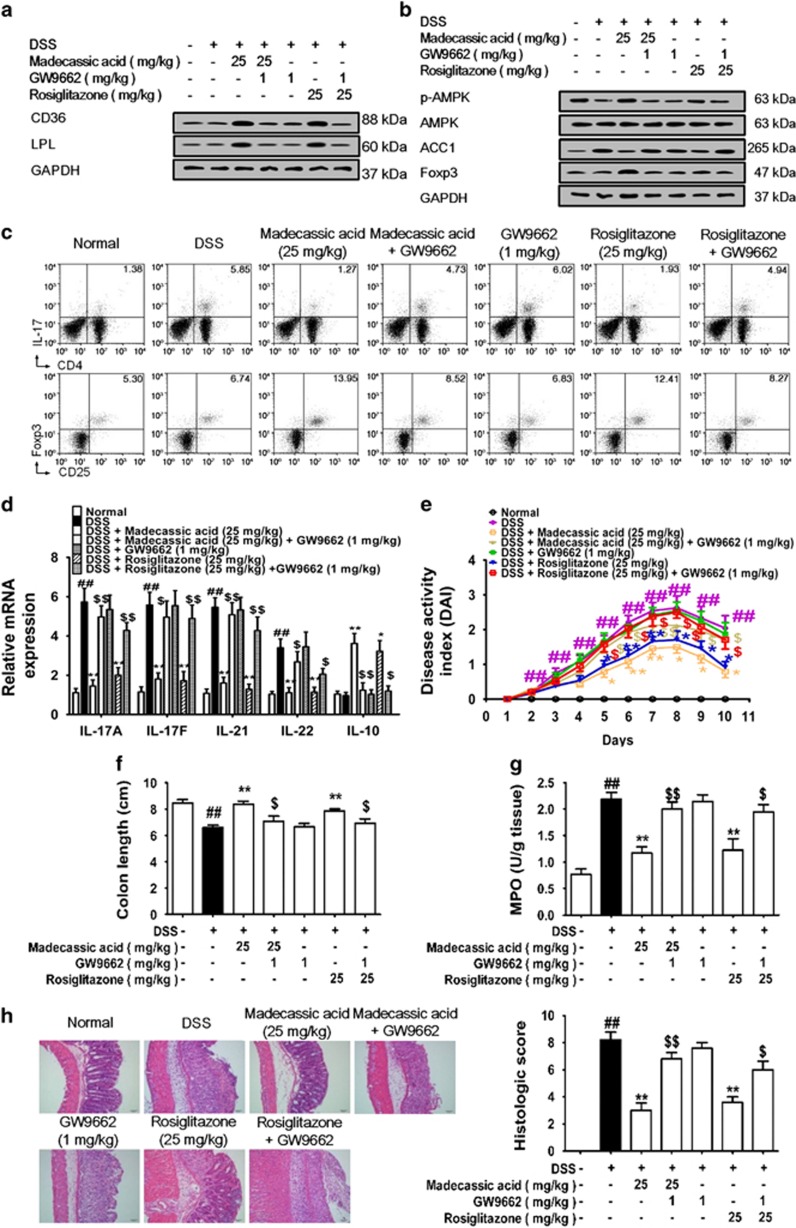
Madecassic acid recovered T helper type 17 (Th17)/regulatory T (Treg) balance in DSS-induced mice colitis through the PPAR*γ*/AMPK/ACC1 pathway. Mice were fed 2.5% DSS for 7 days and then normal water for 3 days. Madecassic acid (25 mg/kg) and rosiglitazone (25 mg/kg) were orally administered for 10 days. GW9662 (1 mg/kg) was administered by an intraperitoneal injection. (**a**) The expression of CD36 and LPL was analyzed by western blot. (**b**) The expression of p**-**AMPK, ACC1 and Foxp3 was detected by western blot. (**c**) The percentages of Th17 and Treg cells were measured by flow cytometry. (**d**) The levels of IL**-**17A, IL**-**17F, IL**-**21, IL**-**22 and IL**-**10 were assessed by real**-**time PCR. (**e**) DAI scores. (**f**) Colon length. (**g**) MPO activity. (**h**) H&E scores. GAPDH was used as a cytoplasm marker. The data were expressed as means±S.E.M., *n*=5–8. ^#^*P*<0.05, ^##^*P*<0.01 *versus* normal group; *P<0.05, **P<0.01 *versus* DSS group ^$^*P*<0.05, ^$$^*P*<0.01 *versus* madecassic acid group

To further ascertain the causal link between PPAR*γ* activation, AMPK/ACC1 signaling regulation, Th17/Treg balance restoration and colitis amelioration by madecassic acid, madecassic acid was orally administered in combination with GW9662 in mice fed with DSS. GW9662 counteracted the madecassic acid**-**induced enhancement of CD36 and LPL expression in the colons (Figure 8a and Supplementary Figure S1). As expected, madecassic acid promoted the activation of AMPK and inhibited the expression of ACC1 in the colons, and these effect were markedly weakened by GW9662 (Figure 8b and Supplementary Figure S1). The decreased percentage of Th17 cells and the increased percentage of Treg cells induced by madecassic acid in the colons were reversed by GW9662 (Figure 8c and Supplementary Figure S1). Moreover, GW9662 weakened the downregulation of Th17 cell**-**specific cytokine expression and the upregulation of IL**-**10 expression by madecassic acid in the colons (Figure 8d) and diminished madecassic acid**-**induced upregulation of Foxp3 expression (Figure 8b and Supplementary Figure S1) and protection against colitis (Figures 8e–h). These results revealed that madecassic acid exerted anti**-**colitis effect by restoring the Th17/Treg balance by shifting Th17 toward Treg cells via the PPAR*γ*/AMPK/ACC1 pathway.

## Discussion

Four main pentacyclic triterpenes in *C. asiatica* could alleviate DSS**-**induced colitis in mice by oral administration. Among them, madecassoside conferred superior protection against the pathological lesions in the colon. When rectally administered, madecassoside but not madecassic acid was unable to protect against colitis. As madecassoside was the most abundant triterpene in *C. asiatica* and it would rapidly dehydrate into madecassic acid in the intestines, we postulated that madecassoside was the primary active ingredient of *C. asiatica* for ameliorating colitis, and it functioned through madecassic acid.

DSS application results in mucosal barrier damage with associated microbial translocation, inflammation and immune activation.^31^ The excessive activation of the immune system can be primarily attributed to Th17/Treg imbalance, resulting from the increase of functionally active Th17 cells and a lack of immune**-**suppressive Treg cells.^32, 33, 34^ Thus the restoration of Th17/Treg balance is conducive to the immune homeostasis and may be an effective therapeutic strategy for UC. In this study, madecassic acid recovered the Th17/Treg balance by decreasing the percentage of Th17 cells and promoting the expansion of Treg cells without affecting the percentages of Th1 and Th2 cells in mice fed with DSS.

Restoring the Th17/Treg balance could be achieved by inhibiting Th17 differentiation, promoting Treg differentiation and shifting between Th17 and Treg cells.^35, 36, 37, 38^ *In vitro*, madecassic acid decreased the proportion of CD4^+^IL**-**17^+^ T cells and markedly increased the proportion of CD4^+^Foxp3^+^ T cells and the expression of Foxp3 and IL**-**10 under Th17**-**polarizing conditions. However, it did not affect the differentiation of Treg cells under Treg**-**polarizing conditions. It was suggested that madecassic acid might restore Th17/Treg balance by enhancing the shift of Th17 toward Treg cells.

The shift of Th17 toward Treg cells can be induced by energy metabolism regulators.^39, 40, 41^ Activated T cells experience an enormous metabolic switch to meet the demands of cell polarization and implementation of their function.^42, 43^ Downregulation or deletion of ACC1 inhibits the plasticity of human and mouse Th17 cells, characterized by promoting the shift of Th17 toward Treg cells.^27^ Madecassic acid was shown to enhance the shift of Th17 toward Treg cells by downregulating the expression of ACC1, and it would lose the ability in the presence of oleic acid, suggesting that ACC1**-**mediated *de novo* fatty acid synthesis was critical for madecassic acid**-**mediated Th17/Treg rebalance.

The activation of AMPK, an upstream regulator of ACC1, will reduce Th17 cell differentiation and favor Treg cell development.^44, 45, 46^ In this study, madecassic acid was shown to accelerate AMPK activation, and compound C abolished madecassic acid**-**induced activation of AMPK and downregulation of ACC1 expression under Th17**-**polarizing conditions, indicating that madecassic acid downregulated the ACC1 expression by activating AMPK in Th17 cells. Moreover, both compound C and AMPK siRNA reversed the shift induced by madecassic acid. These findings revealed that madecassic acid promoted an ACC1**-**mediated shift of Th17 toward Treg cells via activating AMPK under Th17**-**polarizing conditions.

PPAR*γ*, a ligand**-**dependent nuclear receptor that has vital roles in adipogenesis, glucose metabolism and immune modulation, is highly expressed in the colon.^47, 48, 49^ Intensive preclinical studies have highlighted that the activation of PPAR*γ* conferred protection against colitis in mice.^50, 51, 52^ Madecassic acid was shown to activate AMPK in a PPAR*γ***-**dependent manner and induce the AMPK/ACC1**-**mediated shift of Th17 toward Treg cells by activating PPAR*γ*. In contrast, inhibition or depletion of AMPK did not inhibit madecassic acid**-**induced translocation of PPAR*γ* into the nucleus. The findings suggested that AMPK might be a downstream effector of PPAR*γ*.

There are many reports which indicate that high**-**affinity synthetic ligands of PPAR*γ*, such as thiazolidinediones, have immunoprotective roles in experimental colitis.^53, 54, 55^ Similar to rosiglitazone, madecassic acid promoted the expression of PPAR*γ***-**responsive genes CD36 and LPL, induced PPAR*γ* translocation from cytoplasm to nucleus and the binding of PPAR*γ* to a reporter gene, which could be diminished by PPAR*γ* antagonists or PPAR*γ* siRNA. These results indicated that madecassic acid acted as a PPAR*γ* agonist in regulating the AMPK/ACC1**-**mediated Th17/Treg balance.

In conclusion, madecassoside was the major active ingredient of *C. asiatica* in alleviating DSS**-**induced colitis, and it acted through the intestinal metabolite madecassic acid. Madecassic acid exerted anti**-**colitis effect by restoring Th17/Treg balance via the PPAR*γ*/AMPK/ACC1 pathway.

## Materials and methods

### Reagents

Madecassoside (C48H78O20, molecular weight (MW): 975.12, purity≥98%), asiaticoside (C48H78O19, MW: 959.12, purity≥98%), madecassic acid (C30H48O6, MW: 504.70, purity≥98%) and asiatic acid (C30H48O5, MW: 488.70, purity≥98%) purchased from Zelang Pharmaceutical Technology Co., Ltd (Zelang, Nanjing, China) were suspended in 0.5% sodium carboxy methyl cellulose (CMC**-**Na) for oral or rectal administration. Madecassic acid was dissolved in dimethyl sulfoxide (DMSO) and diluted to 10 mM stock solution in phosphate-buffered saline for *in vitro* studies. Cyclosporin A was received from Ipsen Pharma (Ethypharm, Houdan, France). DSS (MW: 36**-**50 kDa) was obtained from MP Biomedicals Inc. (Irvine, CA, USA). Mouse IL**-**17, IL**-**21, IL**-**22 and IL**-**10 ELISA kits were purchased from Dakewe Biotech Co., Ltd (Shenzhen, China). MPO activity assay kit was obtained from Nanjing Jiancheng Bioengineering Institute (Jiancheng, Nanjing, China). Murine IL**-**6, IL**-**23, IL**-**1*β* and human IL**-**2, TGF**-***β*1 were provided by R&D Systems (Minneapolis, CA, USA). Mouse CD4^+^ CD62L^+^ T Cell Isolation Kit was purchased from Miltenyi Biotec Inc. (Santa Barbara, CA, USA). EL**-**4 mouse lymphoma cell line was provided by American Type Culture Collection (ATCC, Manassas, VA, USA). FITC**-**anti**-**CD4, PE**-**anti**-**IL**-**17A, PE**-**anti**-**CD25, APC**-**anti**-**IL**-**4, PE**-**anti**-**IFN**-***γ* and APC**-**anti**-**Foxp3 were purchased from eBioscience, Inc. (Affymetrix, San Diego, CA, USA). Purified anti**-**mouse CD3*ɛ* and anti**-**mouse CD28 antibodies were provide by BD Biosciences, Inc. (San Jose, CA, USA). GW9662 and T0070907 (PPAR*γ* antagonists), 5**-**aminoimidazole**-**4**-**carboxamide1**-***β***-**D**-**ribofuranoside (AICAR, AMPK activator), 5**-**(tetradecyloxy)**-**2**-**furoic acid (TOFA, ACC1 inhibitor), citric acid (ACC1 activator), oleic acid (ACC1 catalytic product), compound C (AMPK inhibitor), rosiglitazone (PPAR*γ* agonist) and cycloheximide (CHX, protein synthesis inhibitor) were purchased from Sigma Aldrich (St. Louis, MO, USA). Act**-**D (RNA polymerase inhibitor) was purchased from Zhejiang Haizheng Pharmaceutical Co. Ltd (Haizheng, Hangzhou, China). TRIzol, Dye DAPI and Lipofectamine 2000 reagents were purchased from Invitrogen (Carlsbad, CA, USA). ACC1 siRNA, AMPK siRNA and PPAR*γ* siRNA (m) were supplied by Santa Cruz Biotechnology (Santa Cruz, CA, USA). Luciferase Reporter Gene Assay Kit was from Promega Biotech Co., Ltd. (Madison, WI, USA). LanthaScreen TR**-**FRET PPAR*γ* Competitive Binding Assay was purchased from Thermo Fisher Co., Ltd (Thermo Fisher, Waltham, MA, USA). Primary antibodies against the following targets: ACC1, p**-**AMPK, AMPK, PPAR*γ*, p**-**STAT3, p**-**STAT5, STAT3, STAT5, Smad3, p**-**Smad3, AKT, p**-**AKT, JAK2, p**-**JAK2, Foxp3, ROR*γ*t, CD36, LPL, Lamin B1 and glycer**-**aldehyde**-**3**-**phosphate dehydrogenase (GAPDH) were purchased from BioWorld (Atlanta, GA, USA). Ace qPCR SYBR Green Master Mix was obtained from Vazyme Co., Ltd (Vazyme, Nanjing, China). Other analytical reagents were obtained from Sino pharm Chemical Reagent Co. Ltd (Nanjing, China).

### Animals

Female C57BL/6 mice, 6**–**8-week**-**old, were purchased from the Comparative Medicine Center of Yangzhou University (Yangzhou, China). The mice were raised in standard animal cages under specific pathogen**-**free conditions in the animal facility at the Central Animal Care Services. All animal experiments were performed in accordance with the Guide for the Care and Use of Laboratory Animals.

### Induction and assessment of DSS-induced colitis and drug administration

Colitis was induced by an administration of 2.5% (w/v) DSS that dissolved in sterile distilled water *ad libitum* for 7 days followed by normal drinking water for 3 days.^56^ Madecassoside (50 mg/kg), asiaticoside (50 mg/kg), madecassic acid (25 mg/kg), asiatic acid (25 mg/kg) and cyclosporin A (25 mg/kg) were orally or rectally administered daily throughout the experiment. GW9662 (1 mg/kg) was administered by an intraperitoneal injection daily for 10 days. Body weight, diarrhea and hematochezia were measured daily after the induction of colitis. The DAI was accounted by the average value of the following three elements: (a) weight loss (0=none; 1 =1–5% 2=5–10% 3=10–15% 4=over 15%); (b) diarrhea scores (0=normal; 2=loose stools; 4=diarrhea); and (c) blood stool scores (0=normal; 2=hemoccult; 4=gross bleeding).

After the induction of colitis with DSS, the colon length was measured and then the colon was fixed in 4% PBS-buffered formaldehyde as a roll and embedded in paraffin. The 5 mm tissue sections were stained with hematoxylin and eosin. Histological scores were graded as follows: (a) severity of inflammation: 0=none; 1=slight; 2=moderate; 3=severe; (b) sites of inflammation: 0=none; 1=mucosa; 2=mucosa and submucosa; 3=transmural; and (c) lesions of crypt: 0=none; 1=basal 1/3 damaged; 2=basal 2/3 damaged; 3=only surface epithelium intact; 4=entire crypt and epithelium lost. The histological score was assessed by average of the three evaluations with a maximal score of 10.

### Mouse T cell cultures

Naive CD4^+^ T cells were isolated *ex vivo* from the spleens and lymph nodes of mice and purified with magnetic beads CD4^+^ CD62L^+^ T Cell Isolation Kit. The purity of the isolated cells was >95%. Naive CD4^+^ T cells were cultured in RPMI 1640, supplemented with 10% FCS, 500 U penicillin** **streptomycin and 50 *μ*M *β***-**mercaptoethanol. For Th17 cell induction, 3**–**4 × 10^5^ naive T cells were cultured for 4 days with plate**-**bound anti**-**CD3*ɛ* (10 *μ*g/ml), anti**-**CD28 (1 *μ*g/ml), anti**-**IFN**-***γ* (5 *μ*g/ml), anti**-**IL**-**4 (5 *μ*g/ml), rhTGF**-***β*1 (2 *μ*g/ml), rmIL**-**6 (5 *μ*g/ml) and rmIL**-**1*β* (50 *μ*g/ml). For Treg cell induction, 2.5 × 10^4^ naive T cells were cultured for 4 days in the presence of plate**-**bound anti**-**CD3*ɛ* (5 *μ*g/ml), anti**-**CD28 (1 *μ*g/ml), rhIL**-**2 (200 U/ml) and rhTGF**-***β*1 (1 *μ*g/ml). On day 2, rhIL**-**2 (200 U/ml) was added.^28, 57^ Citric acid, TOFA, AICAR, compound C, rosiglitazone, oleic acid, GW9662 and T0070907 were added at the beginning of the cultures. The frequencies of Th17 and Treg cells were detected by flow cytometry.

The EL**-**4 cells were cultured at 37 °C under 5% CO_2_ atmospheric condition in RPMI 1640 medium with a supplement of 10% FBS, 100 U/ml streptomycin and 100 U/ml penicillin. The cells (1 × 10^4^ cells/well) were inoculated into six**-**well plates and incubated with various concentrations of madecassic acid (1, 3, 10 *μ*M), GW9662, T0070907 and rosiglitazone for 24 h at 37 °C and 5% CO_2_ and then analyzed by western blot, real**-**time PCR, immunofluorescence, EMSA or luciferase reporter gene assay.

### Enzyme-linked immunosorbent assay

Colonic tissues isolated from DSS**-**treated mice were homogenates, and the supernatants were collected for the detection of IL**-**17A, IL**-**17F, IL**-**21, IL**-**22 and IL**-**10 level with assays by enzyme**-**linked immunosorbent assay kits according to the manufacturer's instructions.

### Intracellular staining and flow cytometry

Cells were harvested from *in vitro* culture or extracted from colonic lamina propria on day 10 after DSS induction for staining of intracellular IFN**-***γ*, IL**-**4, IL**-**17A and Foxp3. For Th1, Th2 and Th17 intracellular staining, the cells were prestimulated with cell stimulation cocktail in 37 °C for 5 h and then stained with FITC**-**anti**-**CD4 for 30 min followed by fixation and permeabilization and subsequently exposed to PE**-**anti**-**IFN**-***γ*, APC**-**anti**-**IL**-**17A and APC**-**anti**-**IL**-**4 antibodies for 1 h. For Treg staining, the cells were cultured with FITC**-**conjugated**-**CD4 and PE**-**conjugated**-**CD25 for 30 min followed by fixation and permeabilization and subsequently stained with APC**-**conjugated**-**Foxp3 antibodies for 1 h. All flow cytometric measurements were conducted on a FACS Calibur (BD Biosciences, San Jose, CA, USA).^57, 58^

### Gene expression analysis

The total RNA from colonic homogenates or cultured cells was extracted with Trizol reagent according to the manufacturer's instructions and then transcribed into cDNA using HiScript RT SuperMix. Then the expression of T**-**bet, GATA**-**3, ROR*γ*t, Foxp3, ACC1, CD36, LPL, IL**-**17A, IL**-**17F, IL**-**21, IL**-**22 and IL**-**10 were analyzed by Ace qPCR SYBR Green Master Mix with MyiQ2 Detection System. Gene expression was normalized to GAPDH and expressed relative to the normal group according to the 2^−ΔΔCt^ method.

### Western blot

Whole**-**cell lysates or proteins were prepared using the lysis buffer. The proteins were separated by 8% SDS**-**PAGE gel and further transferred to polyvinylidene fluoride (PVDF) membranes. After blocking with 5% nonfat milk for 2 h, the PVDF membranes were incubated overnight at 4 °C. Immunoblotting was performed using specific primary antibodies and incubated with goat anti**-**rabbit horseradish peroxidase for 2 h at 37 °C. Detection was visualized using the Odyssey Infrared Imaging System (LI-COR, Inc., Lincoln, NA, USA).^57^

### Transient transfection

The EL**-**4 cells or purified CD4^+^ T cells were inoculated in six**-**well plates (1 × 10^6^ cells/well). PPAR*γ***-**specific siRNA (PPAR*γ* siRNA), ACC1**-**specific siRNA (ACC1 siRNA), AMPK**-**specific siRNA (AMPK siRNA) or non**-**target**-**specific control siRNA (Control siRNA) plasmids were transiently transfected using Lipofectamine 2000 transfection reagent according to the manufacturer's instructions. After transfection, the cells were added with madecassic acid and cultured for 24 or 96 h, respectively, and the proteins or total RNA were harvested for subsequent studies.

### Molecular docking

The agonist**-**bound PPAR*γ* (Protein Data Bank: 4XLD) from the RCSB Protein Data Bank was chosen as the optimal template. The sequence alignment was adjusted according to the GPCR alignment constraints. The best model was generated, sorted by the GBVI/WSA dG score, energy minimized and hydrogens were added using Protonate3D in MOE. The final model was subjected to a binding site analysis and docking study.^59^

### PPAR*γ* competitive binding assay

The LanthaScreen TR**-**FRET PPAR*γ* competitive binding assay was applied according to the manufacturer's protocol. Madecassic acid or rosiglitazone was cultured with GST**-**fused human PPAR*γ***-**LBD, terbium**-**labeled anti**-**GST antibody and a fluorescently labeled PPAR ligand for 3 h in the dark at room temperature. The FRET signal was valued by excitation at 340 nm and emission at 520 nm for fluorescein and 495 nm for terbium. The ability of binding to the PPAR*γ***-**LBD was measured by the downregulation of the 520 nm/495 nm ratio.^59, 60^

### Electrophoretic mobility shift assay

The DNA**-**binding activity of PPAR*γ* was measured by EMSA using a commercial kit. In brief, biotin**-**labeled PPAR*γ***-**specific oligonucleotides were synthesized as the labeled probe according to the manufacturer's instructions. Nuclear extracts were incubated with poly (dI**-**dC), labeled probe and binding buffer at 25 °C for 10 min. The reaction mixtures were then separated with 5% non**-**denatured polyacrylamide gels at 1 mA/cm at 4 °C for 1.5 h and transferred to a PVDF membrane. The biotin end**-**labeled DNA was tested with a streptavidin**-**HRP conjugate and a chemiluminescent substrate. The membrane was finally detected with X**-**ray film and analyzed with the Quantity One software (BD Biosciences, San Jose, CA, USA).^61^

### Luciferase reporter assay

EL**-**4 cells were incubated in 96**-**well plates (1 × 10^4^ cells/ml), and the cells in each well were co**-**transfected with PPRE**-**REPO. A PPRE**-**driven luciferase reporter plasmid was applied for examining specific activation of PPAR*γ* binding to the PPRE. The cells were suspended in fresh culture medium and exposed to madecassic acid in the presence of GW9662 and T0070907, either alone or in a combination for 12 h. Then the cells were lysed, and the supernatants were collected. The luciferase activity was measured by a luciferase assay system and a multimode reader.^62^

### Statistical analysis

The data were presented as the means±S.E.M. from at least three independent experiments. Statistical analysis was performed by using one**-**way analysis of variance followed by Dunnett's test. *P*<0.05 were accepted as a significant difference.
