# Supplementary figures and images for: Madecassic acid, the contributor to the anti-colitis effect of madecassoside, enhances the shift of Th17 toward Treg cells via the PPARγ/AMPK/ACC1 pathway
Source: Cell Death Dis. 2017 Mar 30;8(3):e2723–. doi: 10.1038/cddis.2017.150 (PMC5386545; doi:10.1038/cddis.2017.150)

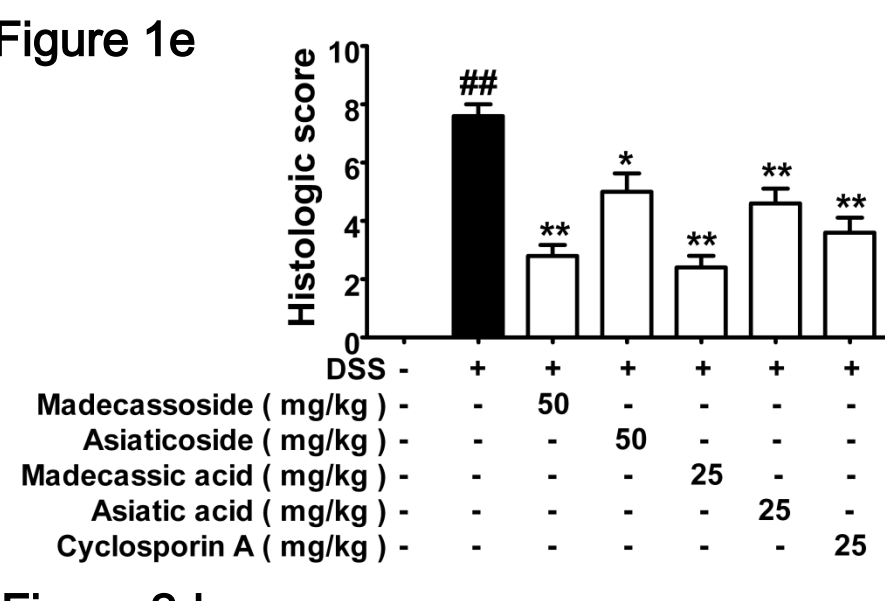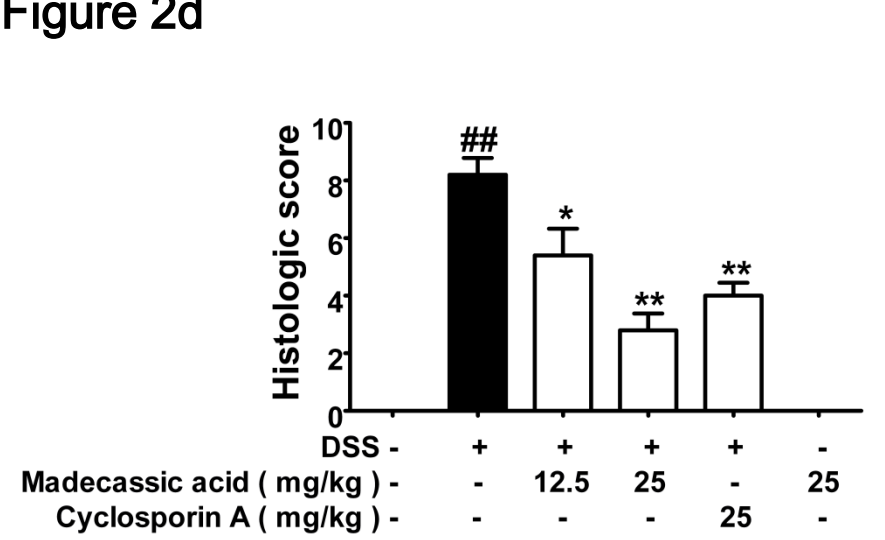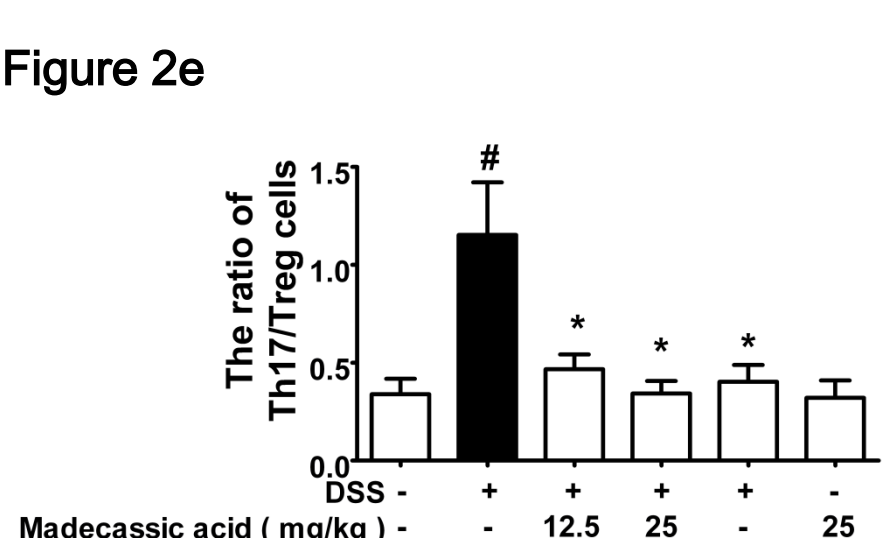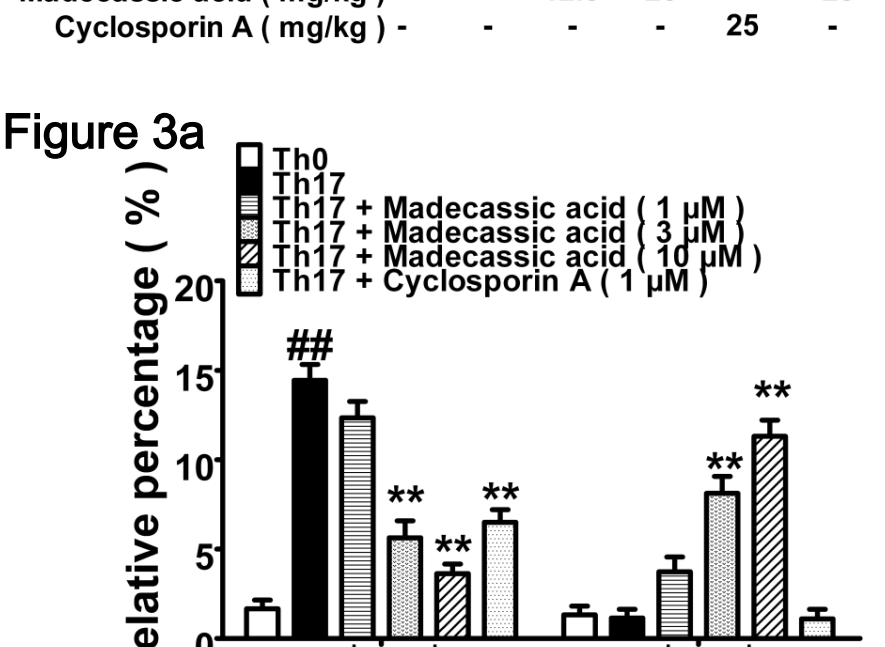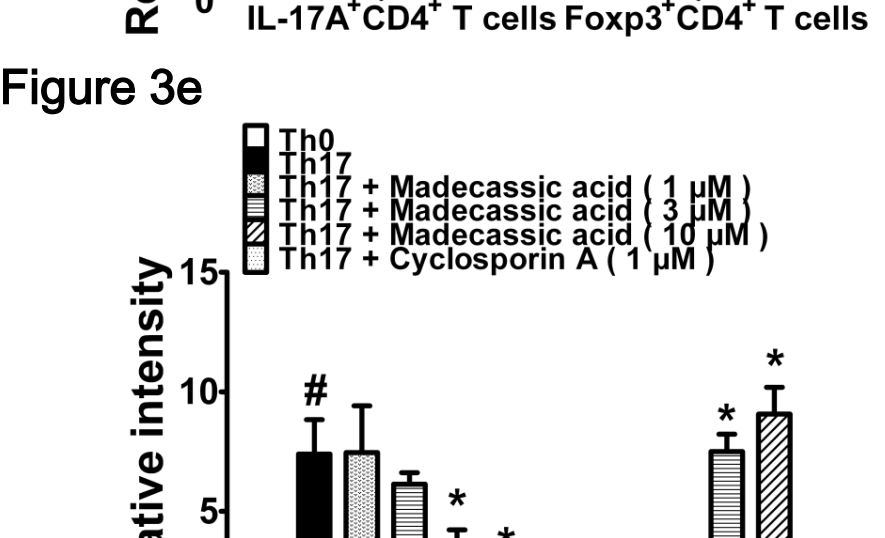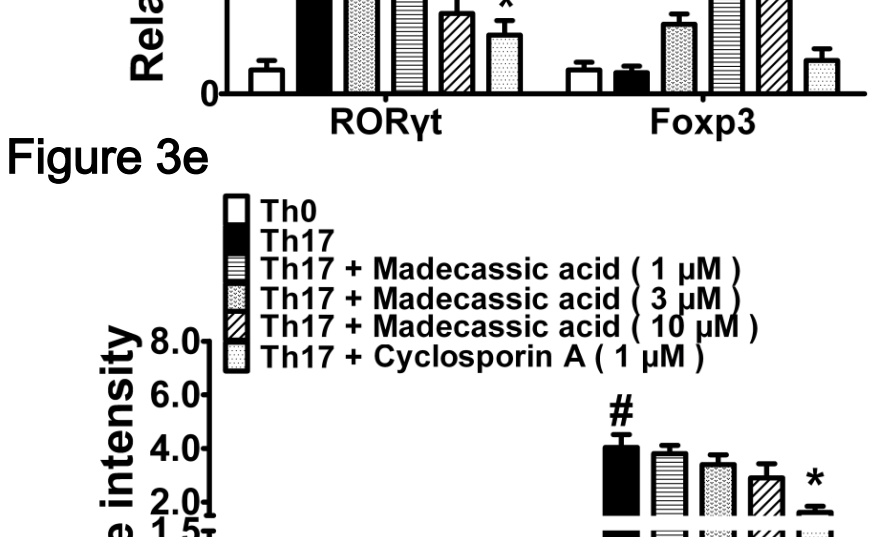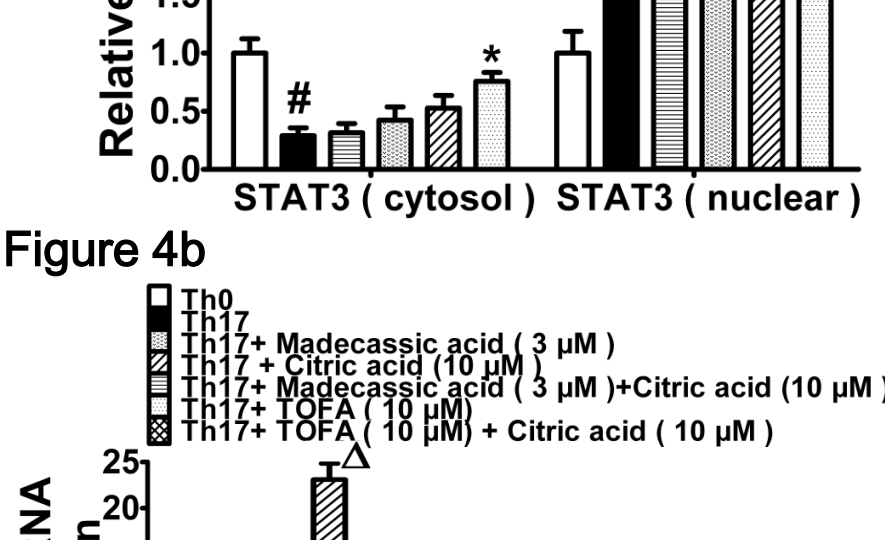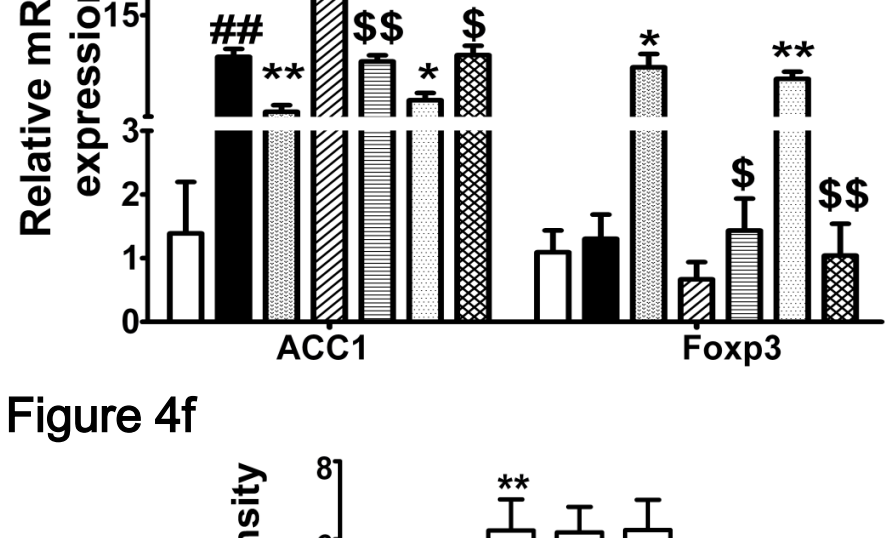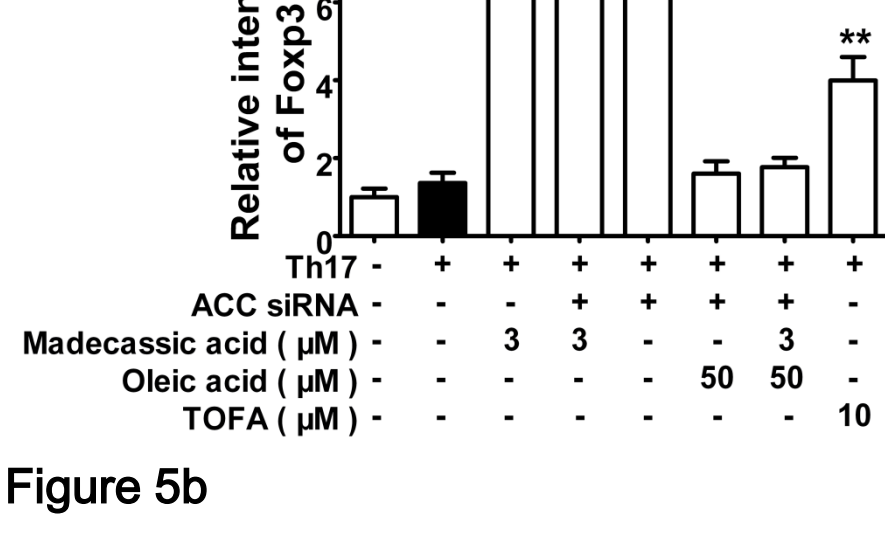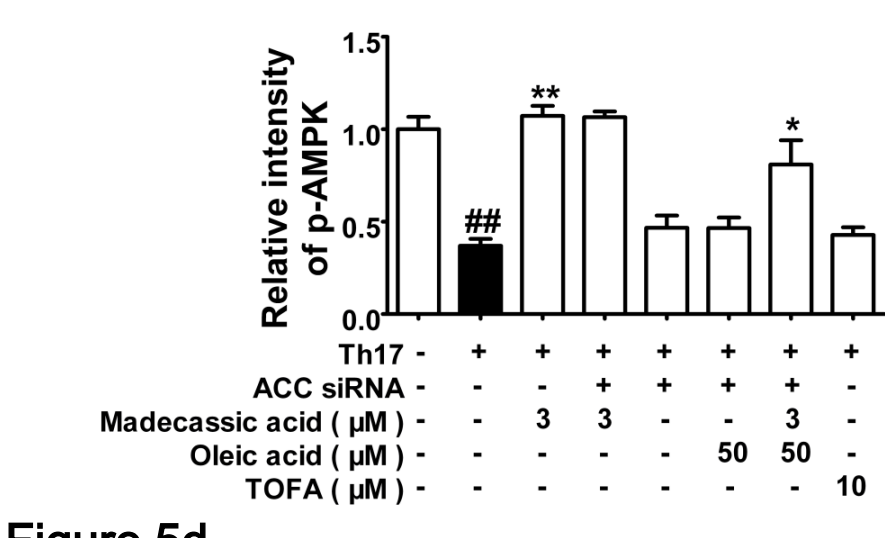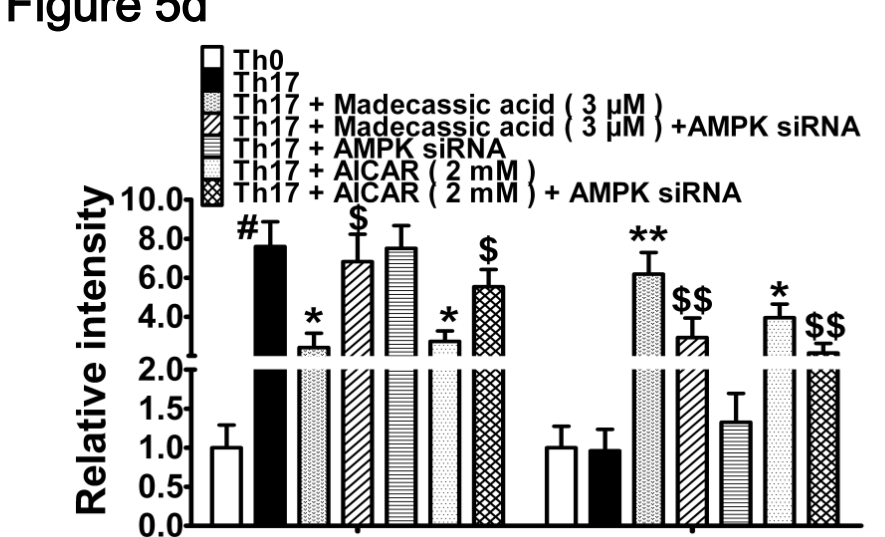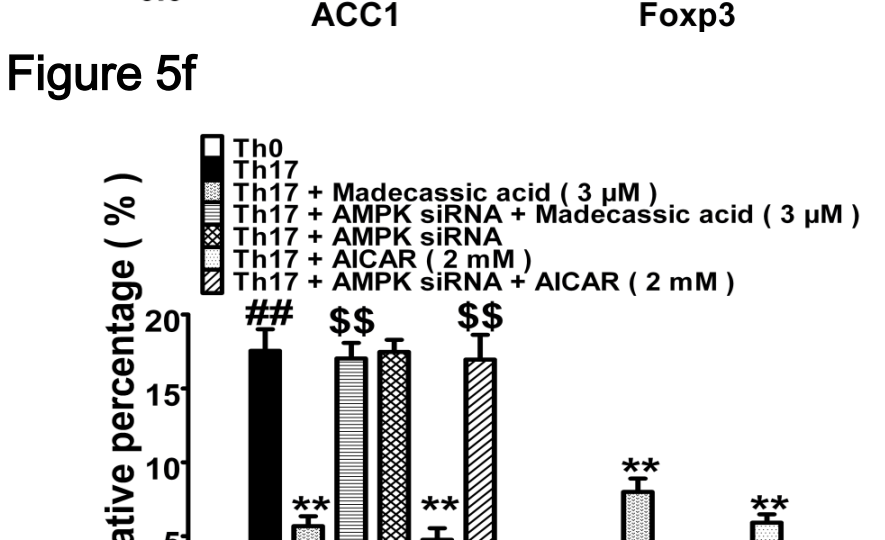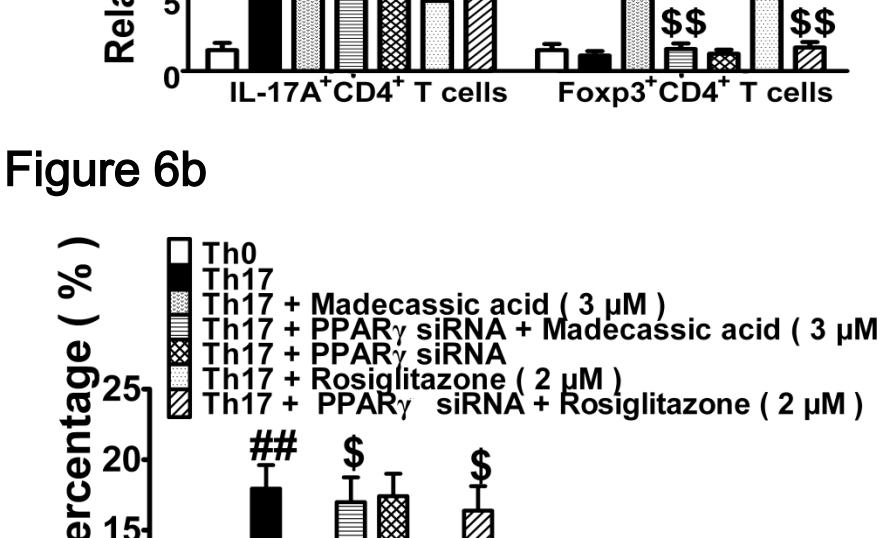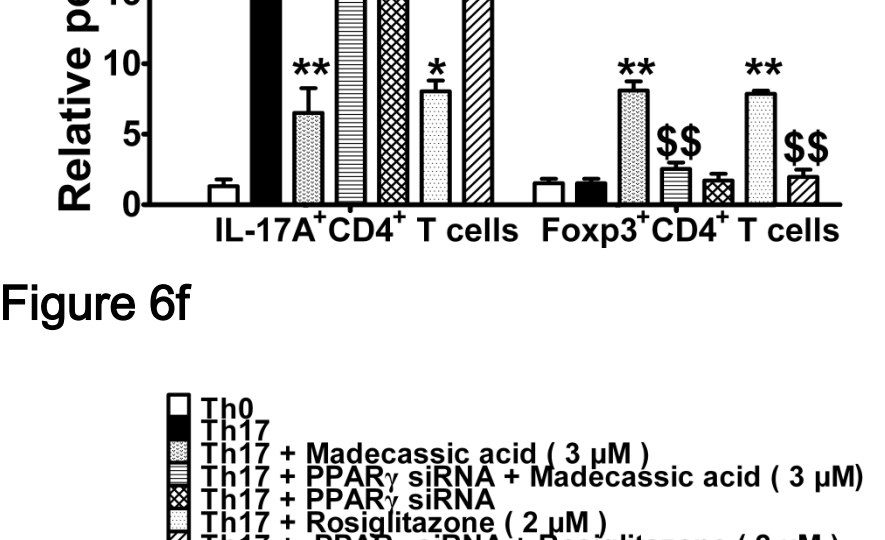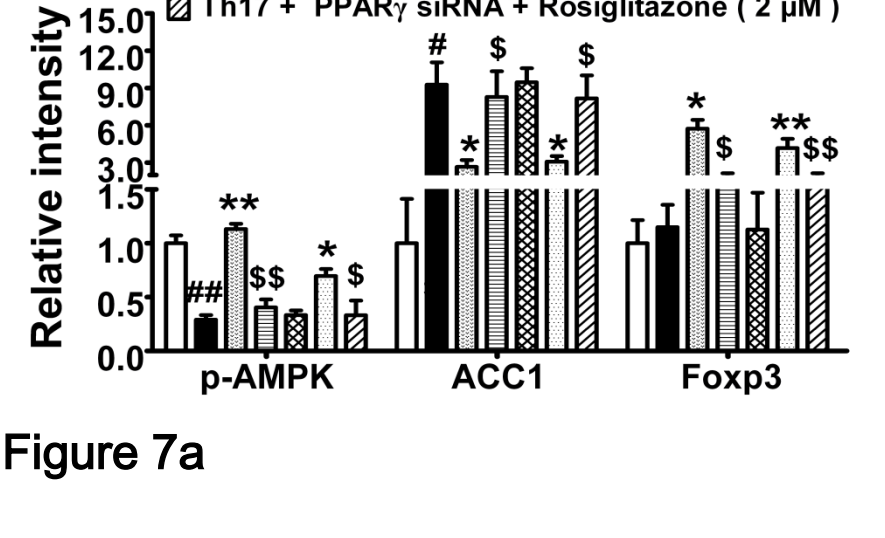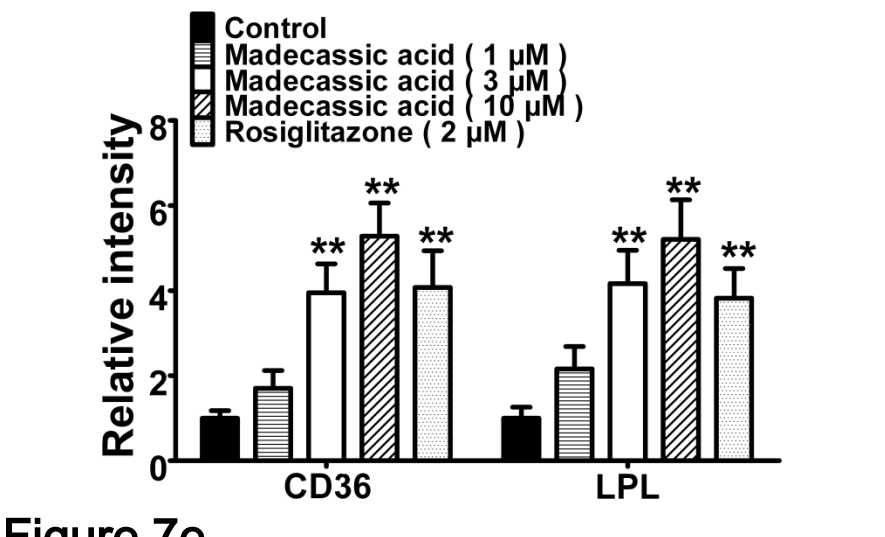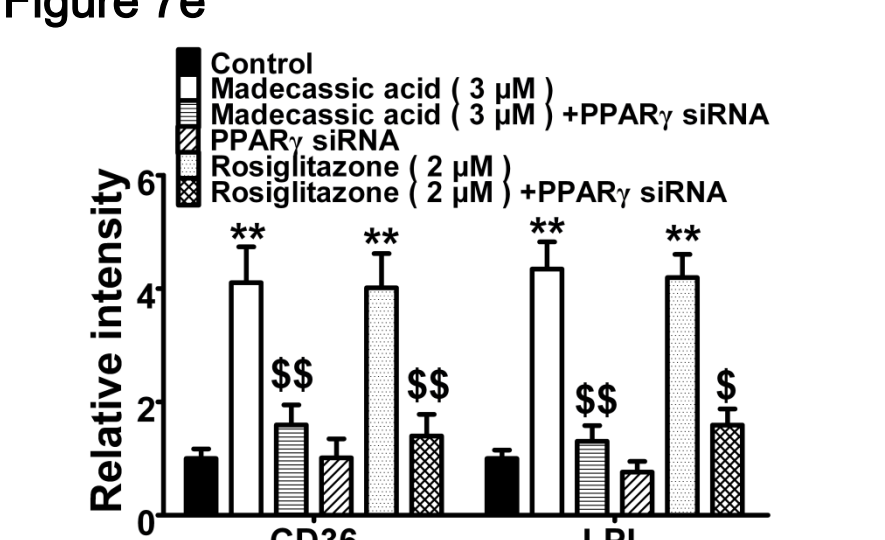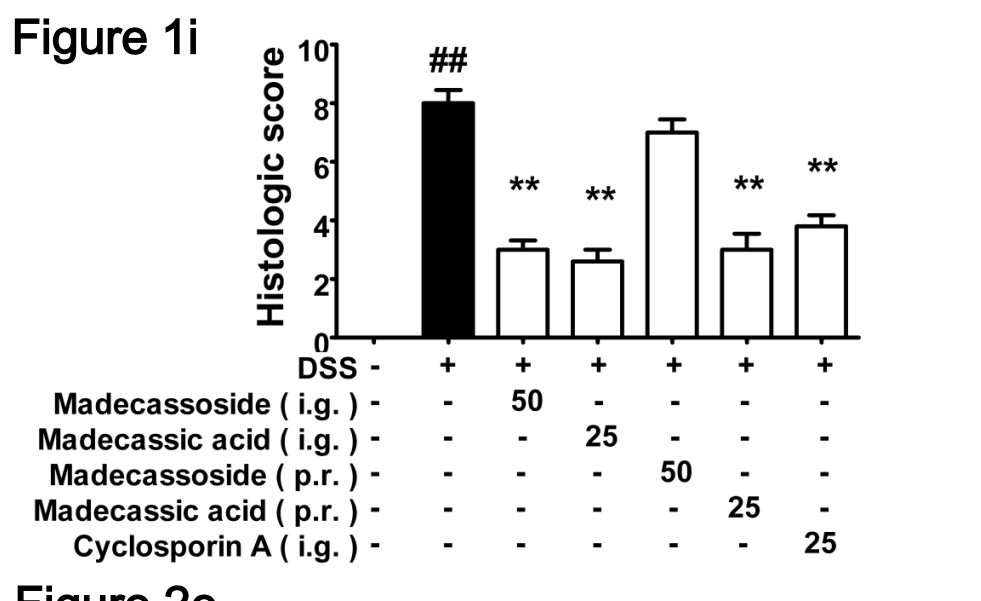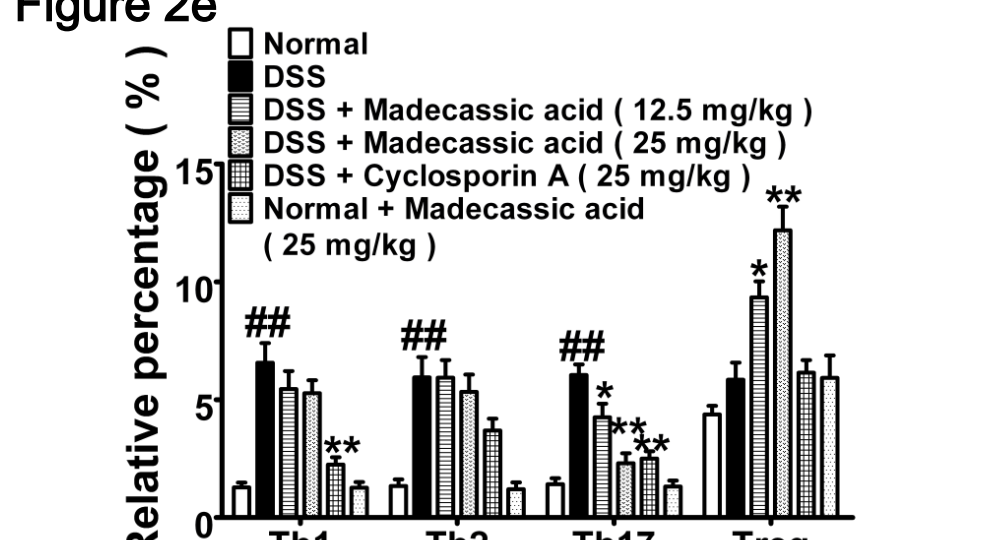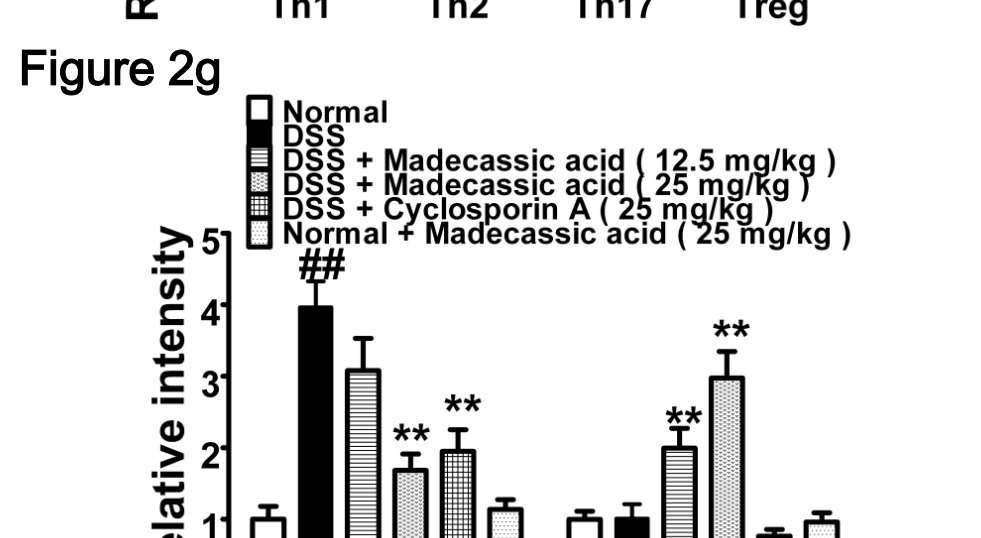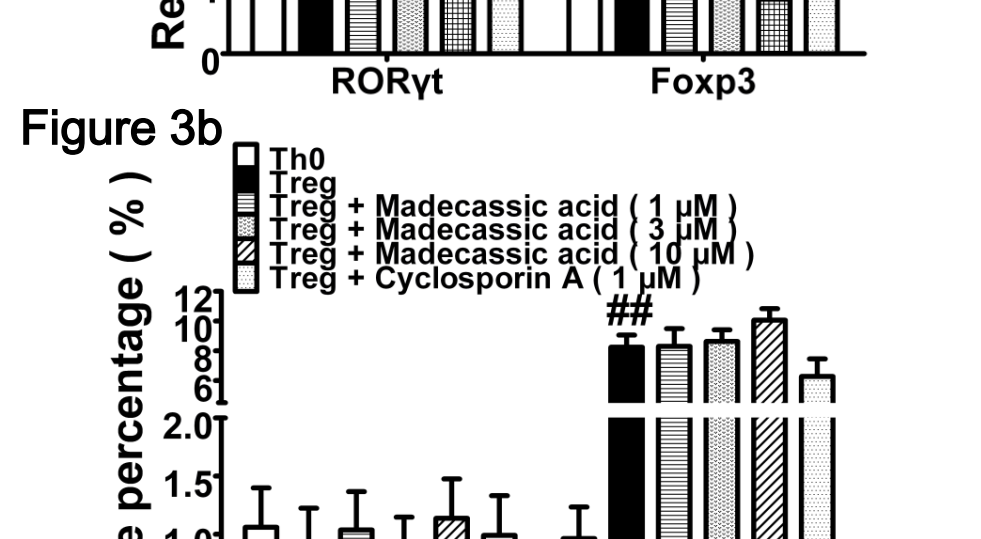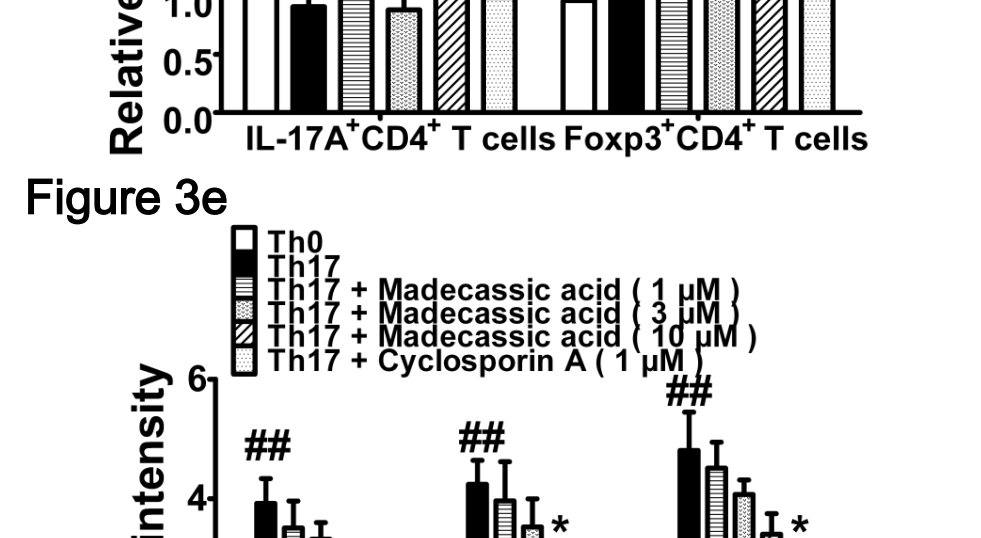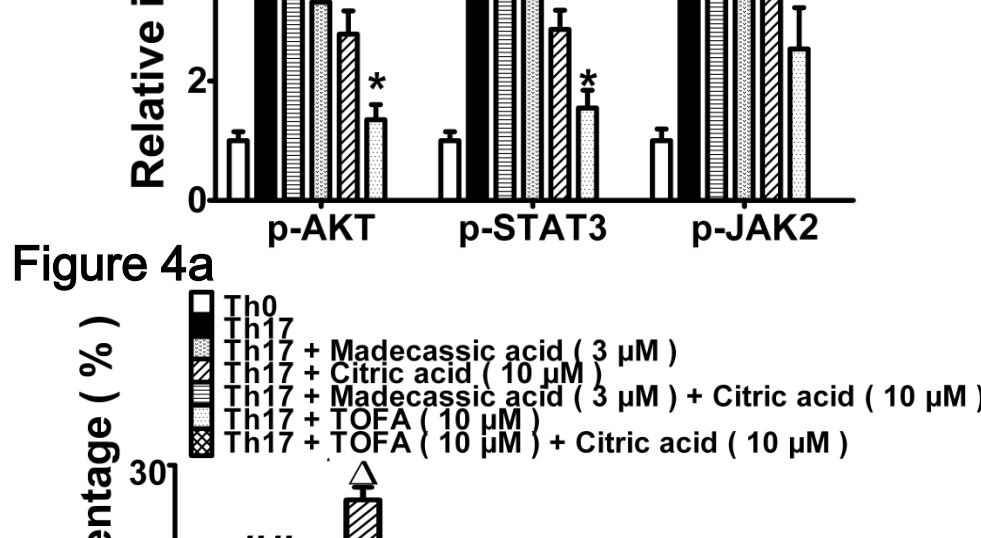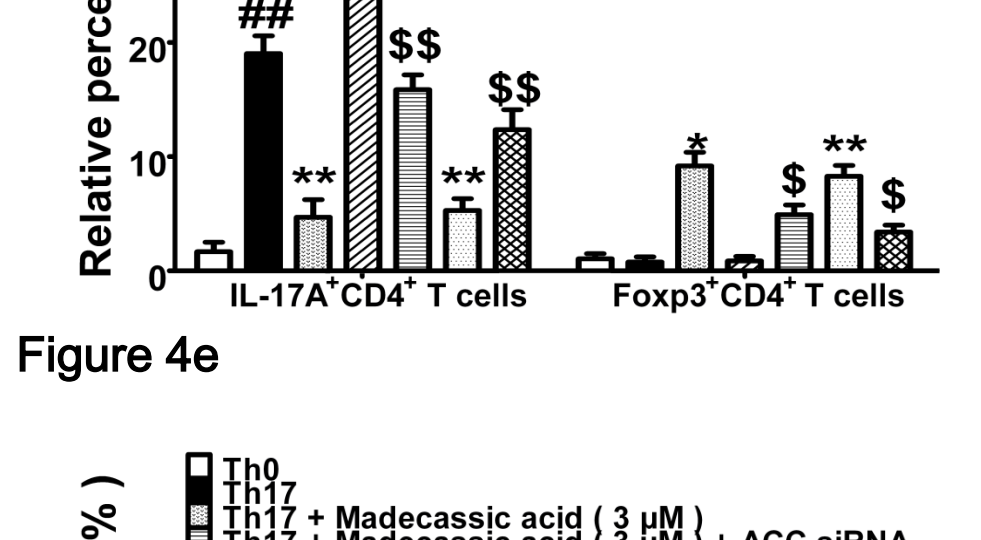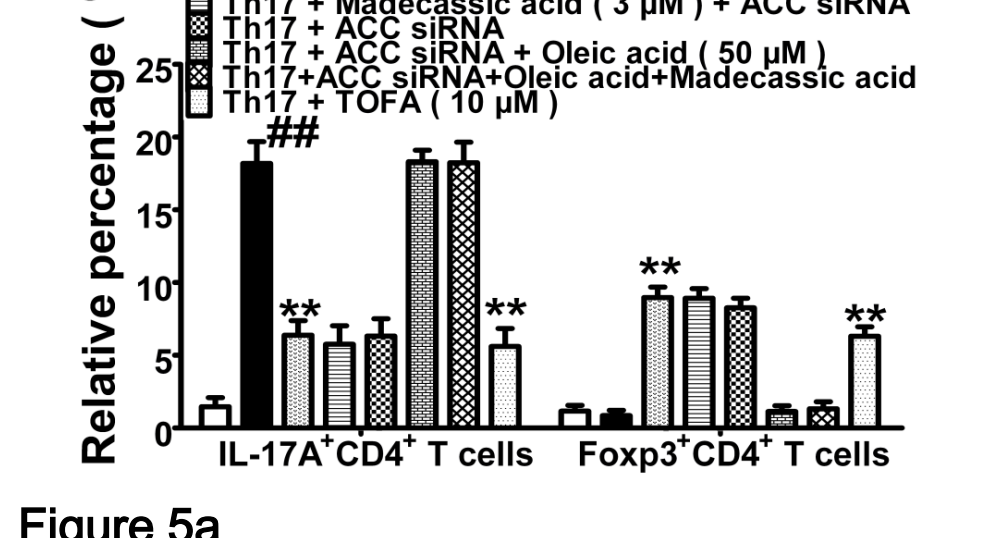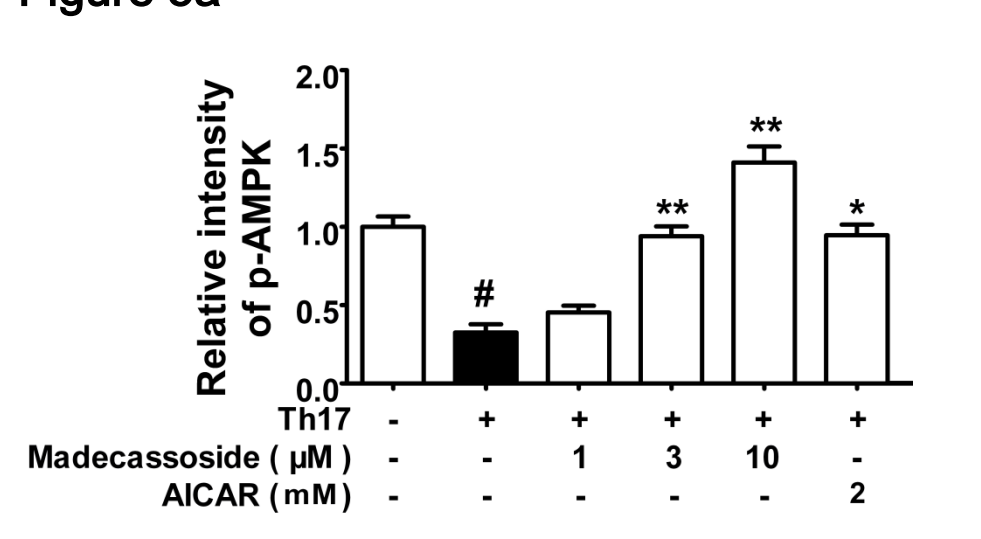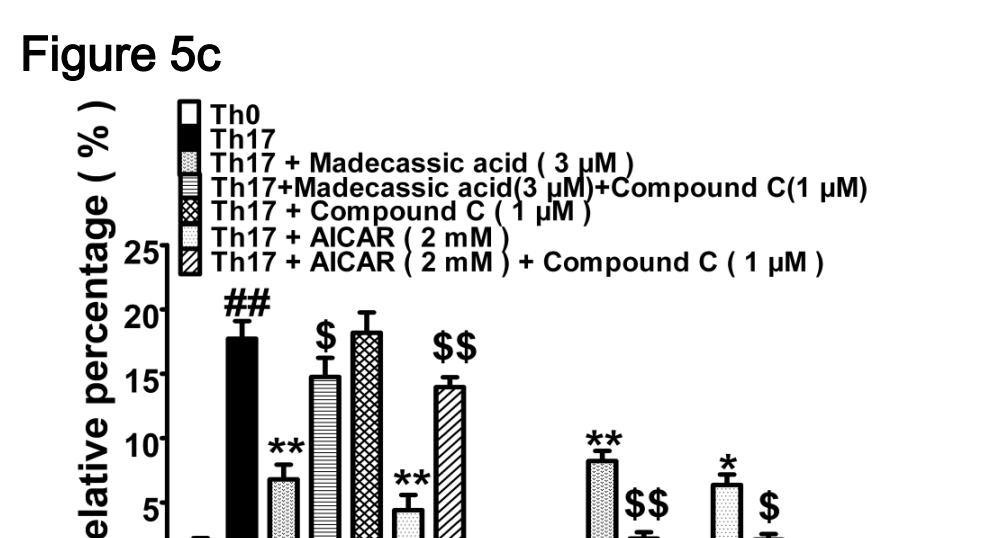

Supplement: Supplementary Information [file cddis2017150x1.pdf]

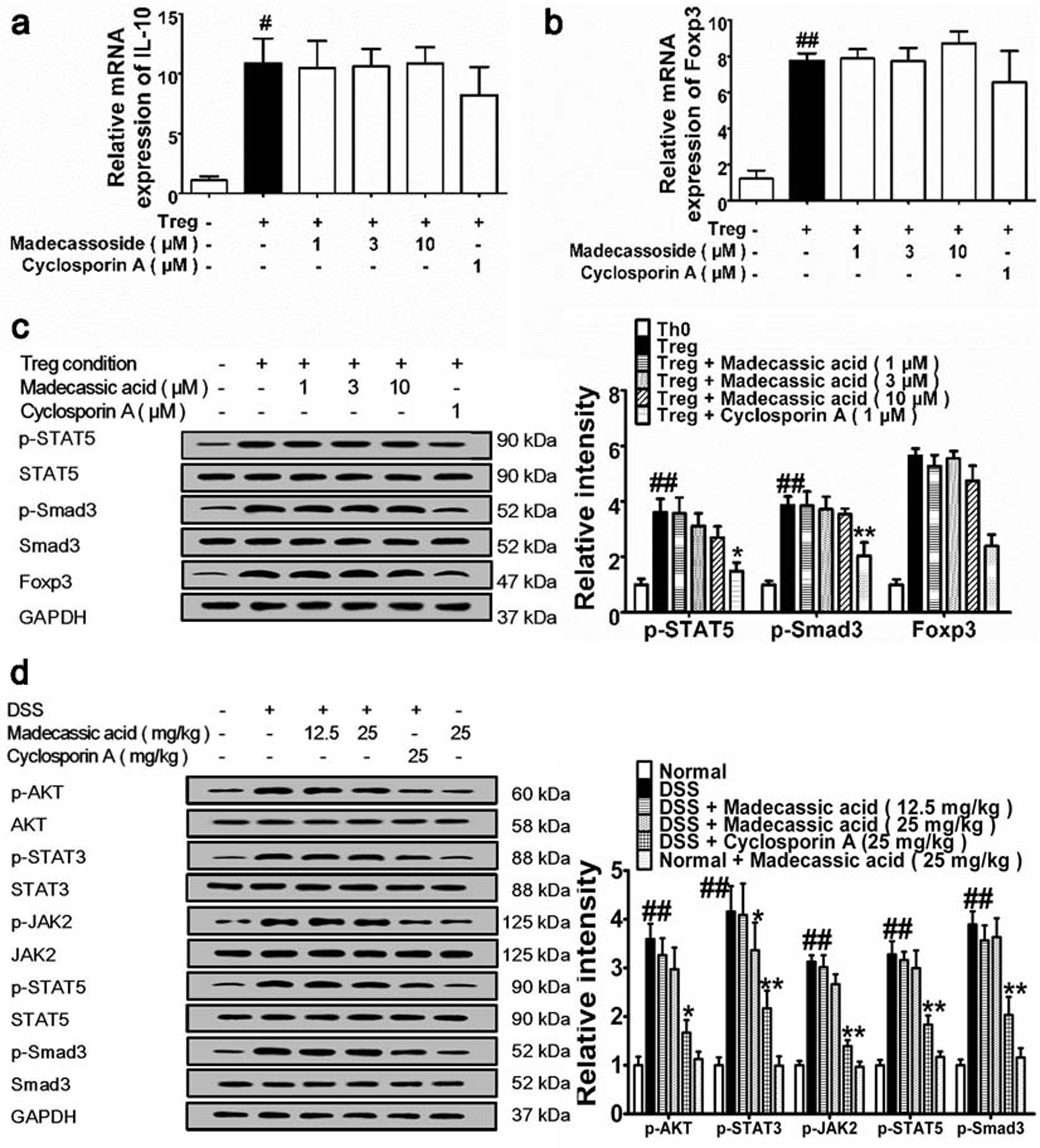

Supplement: Supplementary Figure S1 [file cddis2017150x2.tif]

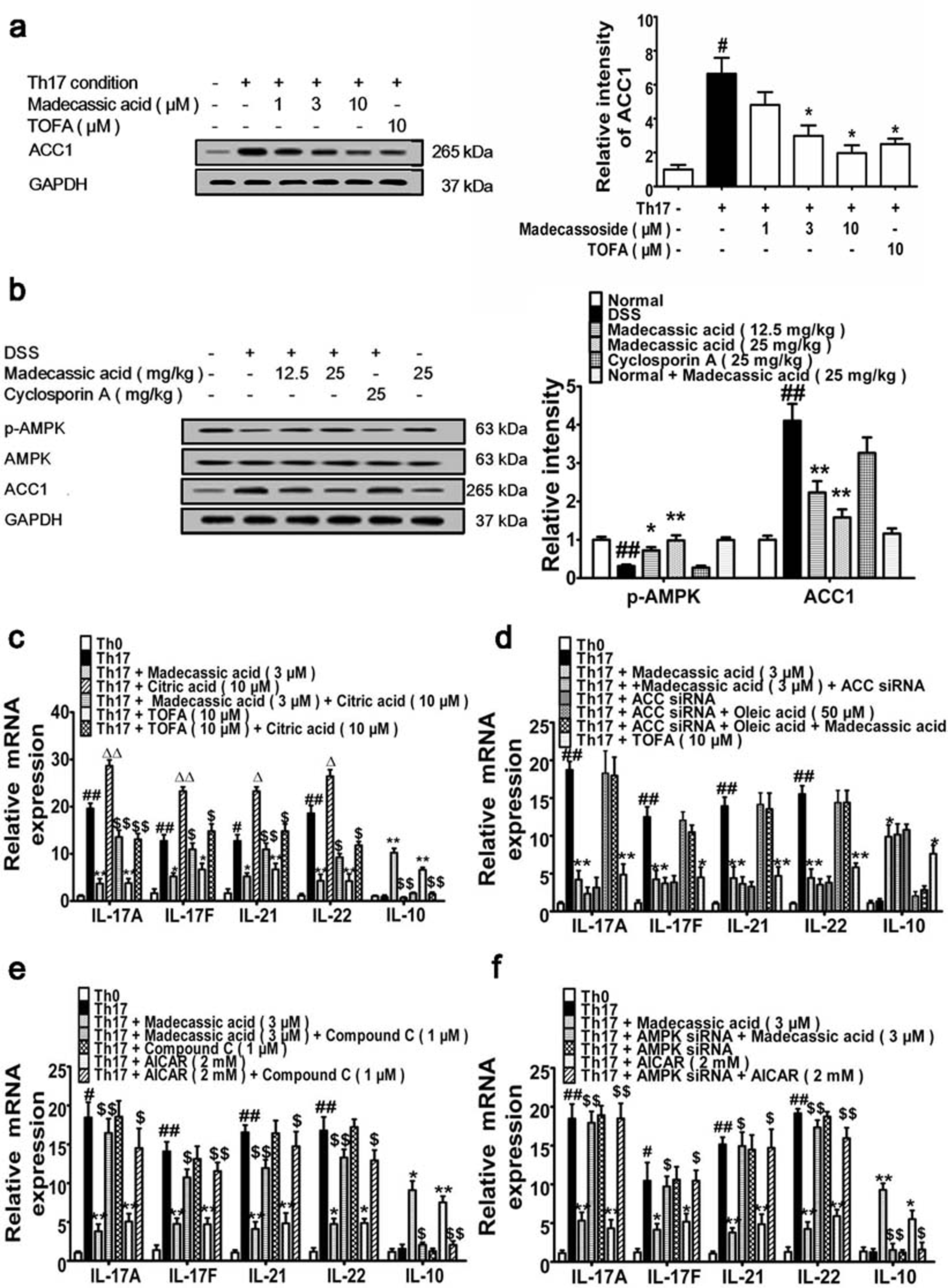

Supplement: Supplementary Figure S2 [file cddis2017150x3.tif]

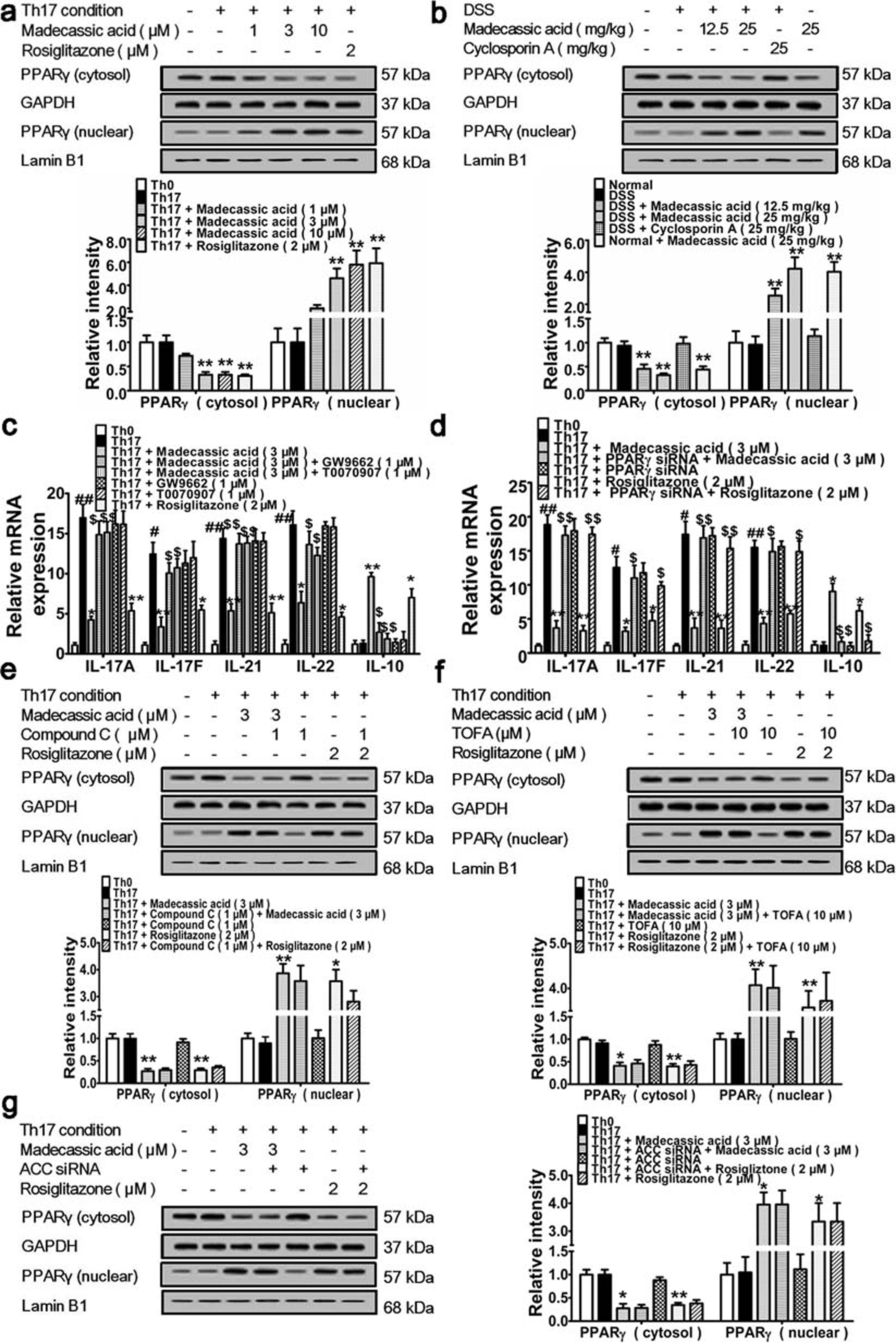

Supplement: Supplementary Figure S3 [file cddis2017150x4.tif]

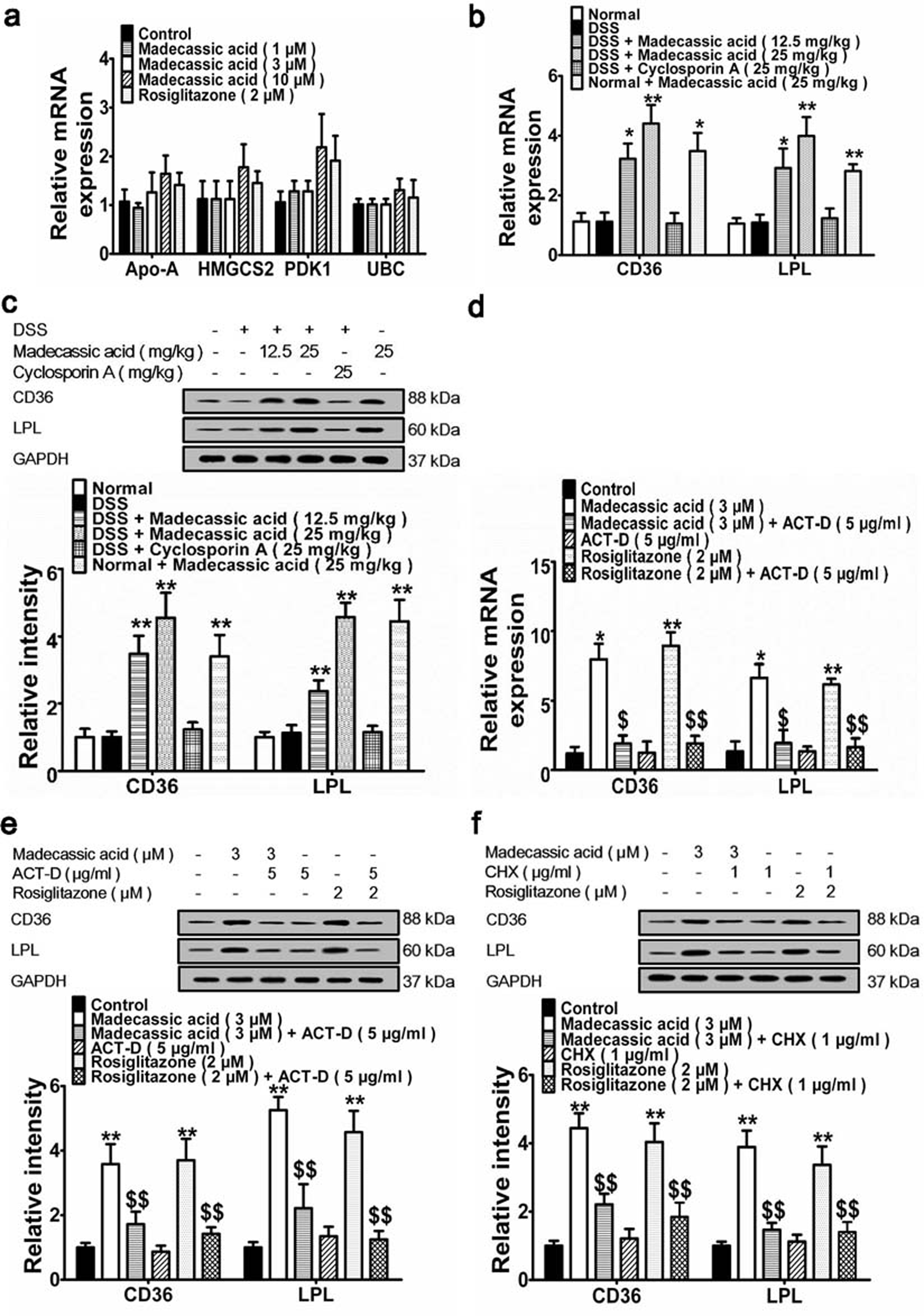

Supplement: Supplementary Figure S4 [file cddis2017150x5.tif]
